# Cross-species transcriptomic analyses reveals common and opposite responses in Arabidopsis, rice and barley following oxidative stress and hormone treatment

**DOI:** 10.1186/s12870-021-03406-7

**Published:** 2022-02-04

**Authors:** Andreas Hartmann, Oliver Berkowitz, James Whelan, Reena Narsai

**Affiliations:** Department of Animal, Plant and Soil Sciences, Australian Research Council Centre of Excellence in Plant Energy Biology, School of Life Sciences, La Trobe Institute for Agriculture and Food (LIAF), La Trobe University, 5 Ring Road Bundoora, Victoria, 3083 Australia

**Keywords:** *Arabidopsis thaliana*, *Oryza sativa*, *Hordeum vulgare*, Arabidopsis, Rice, Barley, Stress, Oxidative, Hormone, Transcriptome, Orthology

## Abstract

**Background:**

For translational genomics, a roadmap is needed to know the molecular similarities or differences between species, such as model species and crop species. This knowledge is invaluable for the selection of target genes and pathways to alter downstream in response to the same stimuli. Here, the transcriptomic responses to six treatments including hormones (abscisic acid - ABA and salicylic acid - SA); treatments that cause oxidative stress (3-amino-1,2,4-triazole - 3AT, methyl viologen - MV); inhibit respiration (antimycin A - AA) or induce genetic damage (ultraviolet radiation -UV) were analysed and compared between Arabidopsis (*Arabidopsis thaliana),* barley (*Hordeum vulgare)* and rice (*Oryza sativa)*.

**Results:**

Common and opposite responses were identified between species, with the number of differentially expressed genes (DEGs) varying greatly between treatments and species. At least 70% of DEGs overlapped with at least one other treatment within a species, indicating overlapping response networks. Remarkably, 15 to 34% of orthologous DEGs showed opposite responses between species, indicating diversity in responses, despite orthology. Orthologous DEGs with common responses to multiple treatments across the three species were correlated with experimental data showing the functional importance of these genes in biotic/abiotic stress responses. The mitochondrial dysfunction response was revealed to be highly conserved in all three species in terms of responsive genes and regulation via the mitochondrial dysfunction element.

**Conclusions:**

The orthologous DEGs that showed a common response between species indicate conserved transcriptomic responses of these pathways between species. However, many genes, including prominent salt-stress responsive genes, were oppositely responsive in multiple-stresses, highlighting fundamental differences in the responses and regulation of these genes between species. This work provides a resource for translation of knowledge or functions between species.

**Supplementary Information:**

The online version contains supplementary material available at 10.1186/s12870-021-03406-7.

## Background

Throughout their lifecycle, plants are exposed to a variety of non-optimal growth conditions lasting from hours and days to seasons. For survival, plants respond to these conditions using adaption as a long-term transgenerational means, acclimation as a medium-term response and/or triggering a stress response for short-term response. The ability to sense and respond to changes in different time spans will ultimately decide if a species survives. Plants are challenged by abiotic and biotic stresses. For biotic stress the perception of microbial pathogens is often by a specific receptor-ligand interaction and thus the ability to recognise a pathogen is often the difference between tolerance and susceptibility [1–3]. For abiotic challenges, from drought, heat and flooding to nutrient or light limitation/excess, the perception and survival can be more complex. For instance, with flooding, the non-optimal condition is multi-factorial where in addition to oxygen limitation, light is also much reduced and the aqueous environment may leave a plant more sensitive to biotic infection [4]. Various proteins that are altered by these sub-optimal conditions can trigger a signalling pathway, are essential for acclimation and have greatly increased our understanding of abiotic stress perception [5]. As plants are exposed to multiple abiotic and biotic stimuli, along with variations in the environment; from soil to beneficial microbial interactions, integration of these various signals is required throughout development to optimise growth [6].

Reactive oxygen species (ROS) have emerged as key signalling molecules for a variety of adverse conditions. ROS play key roles in plant growth and development, thus their signalling role in stress responses integrates plant growth with environmental conditions [7, 8]. The type of ROS produced, and where, defines signalling cascades and how the specificity of signalling is achieved, is clearly an important feature, determined in part by the fact that most ROS species, with the exception of hydrogen peroxide (H_2_O_2_), cannot travel far beyond the site of production. Therefore, their immediate interaction with proteins and metabolites is a feature of how ROS signals are transmitted [8, 9]. Classification of ROS signalling signatures using tools such as ‘ROS wheel’ or ‘Rosmeter’ allows for the comparison of changes in transcriptomic patterns to known perturbations to determine the site of stress perception and signal transduction [9, 10]. At a tissue level, the role of ROS in the regulation of root elongation is detailed to the extent of defining the REDOX sensitive transcription factors (TFs) regulating this process, and perturbation of ROS abundance in root cells alters growth [7, 11, 12]. In response to non-optimal growth conditions, ROS also plays a fundamental role, with apoplastic enzymes such as peroxidases, polyamine oxidases and the respiratory burst oxidase homolog responsible for producing ROS, with the latter considered responsible for the production of ROS from a variety of abiotic and biotic sources [8].

ROS signalling takes place intra- and inter-cellularly, as well as systemically in plants. Peroxisomes, chloroplasts and mitochondria are intra-cellular sources, which constantly produce different ROS species that initiate signalling pathways [13]. While the peroxisomes are a major producer of H_2_O_2_, efficient detoxification systems such as catalase mean that under steady state conditions there is little signal generation as a result [14]. However, inactivation of catalase by salicylic acid (SA) [15], activation by Ca^2+^ [16], and interacting proteins such as NO CATALASE ACTIVITY or NUCLEREDOXIN 1 [17, 18], all regulate the activity of catalase. This shows the complexity of ROS signalling due to the interaction of different pathways, i.e. inactivation of catalase by SA results in an inhibition of both auxin and jasmonic acid (JA) signalling [19]. Chloroplasts produce a variety of ROS, including uniquely singlet oxygen (^1^O_2_) that is inactivated by interaction with carotenoids and other molecules [20]. The interaction with carotenoids produces β-cyclocitral that is involved in retrograde signalling [21]. Both chloroplast and mitochondria produce superoxide (O_2_^.-^) that is rapidly converted to H_2_O_2_ by superoxide dismutase. In both organelles ROS signalling via H_2_O_2_ triggers transcriptional responses with various sensors, mediators and effectors characterised [13]. In the last decade, chloroplasts and mitochondria have emerged as important hubs for sensing and responding via retrograde signalling. Five chloroplast signalling pathways depending on tetrapyrrole, redox/ROS, plastid gene expression, metabolites such as 3′-PHOSPHOADENOSINE-5′-PHOSPHATE, 2-C-METHYL-D-ERYTHRITOL-2,4-CYCLOPYROPHOSPHATE, and dual-located proteins have all been demonstrated to be involved in signalling to different extents [22–24]. Mitochondrial signalling pathways are required for optimal growth and development as disruption of these pathways leads to severely altered growth and stress response phenotypes [25, 26]. Along with a variety of plant hormones that regulate growth and development (auxin, cytokinins) or play role in stress responses (abscisic acid, ABA; salicylic acid, SA; jasmonic acid, JA; ethylene) this leads to a complex network of interacting signalling pathways controlling plant growth and development.

Environmental constraints are predicted to increase over the next decades due to climate change with heat waves, drought periods, water scarcity but also increased duration and frequency of precipitation causing flooding and significantly reducing agricultural productivity with great economic consequences [27–29]. The main focus of crop breeding programs in the past, however, have focused on the maximisation of crop yields, rather than efforts aiming to improve stress tolerance, especially abiotic stress tolerance [30]. The natural adaptation to new environmental conditions is limited by the fast-changing climate [31]. Extensive research efforts are necessary to tackle these issues, hence understanding the plant mechanisms that adjust to adverse environmental conditions is amongst the most significant domains in plant research [32].

In order to increase abiotic stress tolerance in crop species, research has targeted regulatory genes to alter the underlying signalling networks [6] and many TFs have been identified in crop species that confer abiotic stress tolerance ([33, 34] 2019, [35, 36] 2019). Transcription factors are central switches in the regulatory circuitry and represent ideal tools for engineering crop species with enhanced stress tolerance potentially against multiple stresses simultaneously [37]. Advances in functional genomics tools, next-generation sequencing (NGS) technology and the availability of high-quality reference genomes [38–40] for the most important crop species have and will improve the identification of candidate genes, further enabling species comparisons [41]. While organelle signalling has been extensively studies in Arabidopsis as outlined above, and has a large impact on growth and development, little is known about organelle signalling in crop species and if such pathway(s) can confer tolerance to abiotic stress. The identification of NAC013 as a transcription factor that modulates pithiness in *Raphanus sativus* L. (radish), an important agronomic trait, and the variation of this between varieties, point to the potential for organelle signalling pathway to be optimised for agronomic gain [42]. A gene similar to Arabidopsis Radical Cell Death 1 (SRO1) in *Triticum aestivum* enhances growth and tolerance to abiotic stress [43], salt tolerance in *Ipomoea cairica* L. [44], and multiple abiotic stresses in *Oryza sativa* [45]. In Arabidopsis Radical Cell Death 1 has been shown to coordinate mitochondrial and chloroplast activities via interacting with NAC transcription factors that mediate retrograde signalling [46]. Thus, there is the potential to optimise organelle signalling in crop species to increase tolerance to abiotic stress.

In this work, a comparative transcriptome analysis between dicot model, *Arabidopsis thaliana* and the two agronomic monocot species, *Oryza sativa* (rice) and *Hordeum vulgare* (barley), was performed to identify common or opposite responses to a variety of treatments that generate changes in ROS and alter hormones levels, with hormones shown to interact with mitochondrial signalling [47]. Construction of orthogroups [24] in combination with comprehensive gene expression profiling was used to identify commonalities and species-specific differences in a defined biological context.

## Results

### Dynamic expression responses to stress in Arabidopsis, rice and barley

The transcriptome responses of a model species (Arabidopsis) and two crop species (rice and barley) to six treatments designed to mimic stress responses were analysed by using either hormones (abscisic acid - ABA and salicylic acid - SA); treatments that cause oxidative stress (3-amino-1,2,4-triazole - 3AT, methyl viologen - MV); inhibit respiration (antimycin A - AA) or induce genetic damage (ultraviolet radiation -UV) (Table 1). To ensure efficacy of application for the treatments (Table 1) in this study, the expression of marker genes was examined by quantitative reverse transcription polymerase chain reaction (qRT-PCR) in all three species. The starting point for the treatments was the concentration and time that had been previously extensively optimised in Arabidopsis [48–50], and 3 h was the time point at which transcript abundance for the mitochondrial stress marker Alternative oxidase (AOX) peaked. The induction of various marker genes was 16-fold or greater at 3 h in all species and treatments, except in barley, ABA treatment for the marker gene *Beta-glucosidase 31* was > than 8-fold, and with the induction of AOX1a with ABA treatment in barley similar to that seen in rice and Arabidopsis this was considered sufficient. This is consistent with the fact that barley has natural tolerance to a variety of abiotic stresses and has been defined as the most salt tolerant cereal [51] and stress tolerant cereal [52]. Treatment of barley for 6 h with AA and MV did produce similar orders of magnitude of changes in marker genes (Table 2), and thus for these treatments 6 h time point was chosen.

**Table 1:** Overview of treatments used in this study to induce stress response pathways

Treatment	Target	Stress	References
**Antimycin A**	Inhibition of mitochondrial electron transport, cytochrome bc1 complex.	ROS - Oxidative stress	Slater, 1973
**3-amino-1,2,4-triazole**	Inhibition of catalases and carotenoid biosynthesis	ROS - Oxidative stress	Margoliash et al., 1960 Yang et al., 2019 Su et al., 2018
**Methyl viologen**	Competes for electrons with PSI (inhibition of photosynthesis)	ROS - Oxidative stress	Hassan, 1984 Fuerst and Norman, 1991
**Salicylic acid**	SA Receptors	Activation of stress- signalling pathways	Ding et al., 2018 Kaltdorf and Naseem, 2013
**Abscisic acid**	ABA Receptors	Activation of stress- signalling pathways	Ma et al., 2009 Park et al., 2009 Santiago et al., 2009
**Ultraviolet radiation** (UV-C)	DNA, protein and lipids Photosynthesis	DNA damage Photoinhibition	Stapleton, 1992 Gao et al., 2008 Urban et al., 2016

**Table 2:** Gene expression responses of marker genes to validate stress treatments. Transcriptional changes of selected stress marker genes in Arabidopsis, rice and barley. Values represent log2 fold-changes. Details for the references identifying these as markers and full gene descriptions can be seen in ST1

**Arabidopsis**								
gene	AGI	Annotation	3AT (3 h)	AA (3 h)	ABA (3 h)	MV (3 h)	SA (3 h)	UV (3 h)
AOX1a	At3g22370	alternative oxidase 1A	4.85	4.71	1.93	1.78	1.92	3.93
AOX1c	At3g27620	alternative oxidase 1C	−0.1	−0.3	−2	−1.3	−1	0.3
Cys-2	AT5G09570	AT12CYS-2	2.66	4.72	0.22	0.78	−0	3.22
ABF3	AT4G34000	ABA responsive elements-BF3	0.32	0.79	4.04	1.61	0.42	1.38
RD29b	AT5G52300	RESPONSIVE TO DESICCATION 29B	0.34	0.85	9.15	3.73	−0.2	1.06
ERF1	AT3G23240	ethylene response factor 1	3.83	4.1	0.91	5.23	4.13	9.99
PDF1.2	AT5G44420	plant defensin 1.2	1.08	1.38	0	−2	−1.8	3.45
PR-1	AT2G14610	pathogenesis-related gene 1	0.44	1.38	0.53	0.82	7.56	−2
**Rice**								
**gene**	**AGI**	**Annotation**	**3AT (3 h)**	**AA (3 h)**	**ABA (3 h)**	**MV (3 h)**	**SA (3 h)**	**UV (3 h)**
AOX 1A	LOC_Os04g51150	alternative oxidase 1A	6.05	3.28	1.2	3.12	3.01	8.46
AOX 1B	LOC_Os04g51160	alternative oxidase 1B	8.86	5.56	2.73	6.08	4.77	10.9
AOX 1C	LOC_Os02g47200	alternative oxidase 1C	−0.5	−0.5	1.17	0.05	0.05	−1.3
RAB16A	LOC_Os11g26790	Similar to RAB21	0.15	2.19	11.2	0.27	−1.2	1.62
NDB2	LOC_Os05g26660	NADH-ubiquinone oxidoreductase	1.4	1.84	1.25	1.25	1.97	2.82
NDA2	LOC_Os01g61410	NADH-ubiquinone oxidoreductase	10.6	3.55	−0.7	7.26	5.27	10.5
Putative orth.	LOC_Os03g51459	expressed protein (At2g21640 orth.)	4.44	1.89	2.37	0.46	3.44	3.95
**Barley**								
**gene**	**AGI**	**Annotation**	**3AT (3 h)**	**AA (6 h)**	**ABA (3 h)**	**MV (6 h)**	**SA (3 h)**	**UV (3 h)**
AOX1A	HORVU2Hr1G101980	alternative oxidase 1A	6.61	4.79	2.24	0.1	2.19	4.66
Hv_AOX1C	HORVU6Hr1G068150	alternative oxidase 1C	0.2	−0.2	−0.7	−1.2	0.69	−1.3
AOX1D1	HORVU0Hr1G005420	alternative oxidase 1D1	3.84	2.37	−4.5	1.25	−2.7	11.4
AOX1D2	HORVU2Hr1G101990	alternative oxidase 1D2	2.87	1.24	−4.6	−2.6	−2.3	9.97
ABA resp.	HORVU5Hr1G077880	Beta-glucosidase 31	5.58	0.54	3.28	1.51	7.12	1.19
Hv_NDB2	HORVU3Hr1G076920	NAD(P)H-ubiquinone oxidoreductase B3	1.62	4	−2.7	1.94	−0.3	6.24
Hv_NDB3	HORVU7Hr1G073050	NAD(P)H-ubiquinone oxidoreductase B3	2.53	1.93	−1.1	−0.4	0.16	3.71
Stress resp. 3	HORVU5Hr1G087880	Auxin efflux carrier family protein	6.13	2.02	0.19	2.39	4.51	4.17
Stress resp. 4	HORVU0Hr1G019630	expressed protein	6.98	1.95	−0	1.06	4.3	7.3
Stress resp. 6	HORVU5Hr1G097140	PPR-containing protein	NA	NA	1.1	5.53	0.85	0.07

**Fig. 1:**
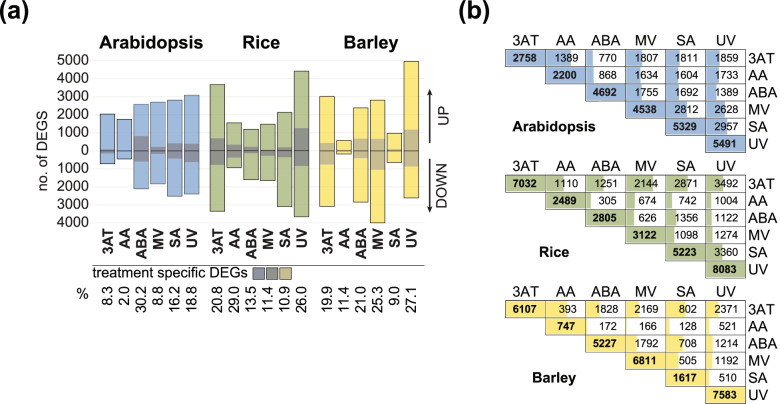
Summary of transcriptome responses to treatments in Arabidopsis, rice and barley. (a) Numbers of differentially expressed genes (DEGs; |log2 (fold change)| > 1, FDR < 0.05) and for each treatment in Arabidopsis (At), rice (Os) and barley (Hv). The number of species-specific DEGs for each treatment are indicated by dark shading; 3AT = 3-amino-1,2,4-triazole, AA = antimycin A, ABA = abscisic acid, MV = methyl viologen, SA = salicylic acid, UV = ultraviolet radiation. (b) Matrix showing the number of overlapping DEGs between treatments (two-way comparisons) in each species

RNA-seq analysis for the response to the different treatments (Table 1) revealed that out of the 19,700, 22,609 and 24,541 detected genes annotated in the Arabidopsis (Tair10), rice (IRGSP-1.0) and barley (IBSCv2) genomes, respectively, a total of 10,462, 13,735 and 14,470 genes were responsive to at least one treatment when compared to the mock treatment (Fig. 1 a; Table S2a-c). For all three species, the largest number of differentially expressed genes (DEGs; FDR < 0.05 and |log2FC| > 1) was observed in response to ultra-violet [53] treatment and smallest number observed in response to antimycin A (AA) treatment (Fig. 1 a). The number of treatment specific DEGs ranged from 2 to 30% in the three species (Fig. 1 a). Twice the number of DEGs were responsive to catalase inhibitor 3-amino-1,2,4-triazole (3AT) in the monocots (rice: 7032 DEGs; barley: 6107 DEGs) compared to the dicot species Arabidopsis (2758 DEGs; Fig. 1 a). The response to 3AT is also more distinct in monocots, with 20.8% in rice and 19.9% in barley of treatment specific DEGs observed in these, compared to 8.3% in Arabidopsis. Notably, the number of DEGs following ABA treatment was more similar between Arabidopsis (4692 DEGs) and barley (5227 DEGs) compared to almost half that number in rice (2805 DEGs) (Fig. 1 a). The ABA response in Arabidopsis was also the most distinct compared to other treatments with 30.2% of all DEGs being treatment specific, while only 2% of AA responsive DEGs were treatment specific. Similarly, the number of DEGs following SA treatment was more similar between Arabidopsis (5329 DEGs) and rice (5223 DEGs) compared to barley (1617 DEGs), which showed around one third that number of DEGs (Fig. 1 a). The response to methyl viologen [54], which leads to oxidative stress and the formation of ROS under illumination in the chloroplast differed between all three species with the largest number of DEGs observed for barley (6811 DEGs), followed by Arabidopsis (4538 DEGs) and rice (3122 DEGs). For each species, two-way comparisons of the number of overlapping DEGs between treatments were carried out, revealing greater conservation overall between responses in Arabidopsis, compared to rice and barley (Fig. 1 b). For example, more than half of all DEGs responsive to AA and 3AT treatment in Arabidopsis overlapped with the other four treatments, with the exception of ABA (Fig. 1 b), while the DEGs responsive to AA and 3AT were more distinct in barley and rice, having a smaller percentage of overlapping DEGs (Fig. 1 b). 3AT and MV responses displayed the greatest overlap in rice, whereas in barley the greatest overlap was observed between 3AT and UV, indicating species-specific responses (Fig. 1 b).

**Fig. 2:**
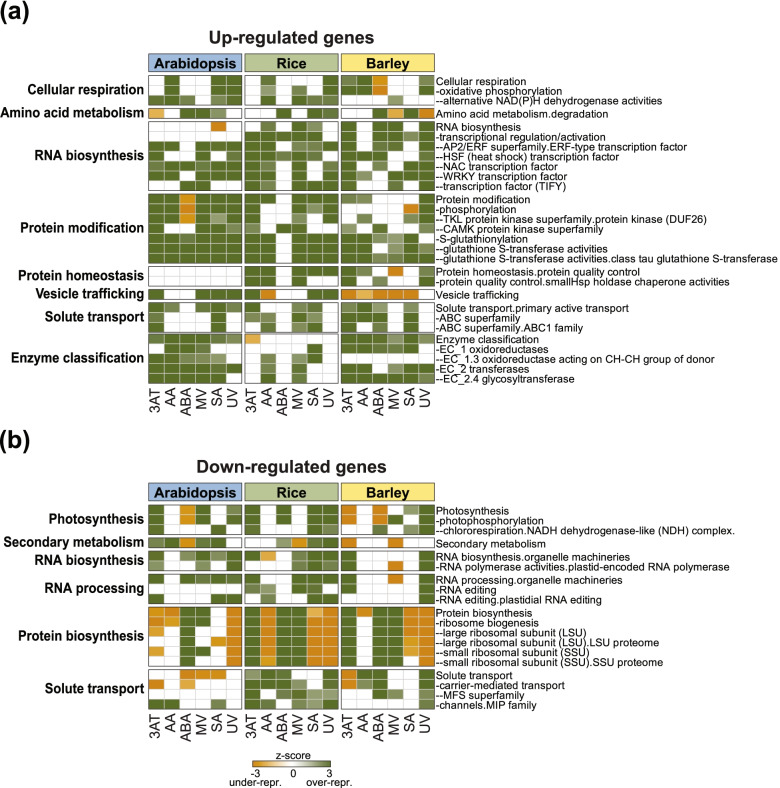
Conserved over-represented functional categories. Analysis of functional categories in each of the species was done using PageMan (Usadel et al., 2006) for (a) up-regulated and (b) down-regulated genes responsive to different treatments in Arabidopsis, rice and barley; 3AT = 3-amino-1,2,4-triazole, AA = antimycin A, ABA = abscisic acid, MV = methyl viologen, SA = salicylic acid, UV = ultraviolet radiation. Only the functional categories that were significantly over-represented (PageMan- ORA-Fisher’s test, *p* < 0.05) in response to at least three stresses and two species are shown

To gain insight into the functions of these DEGs, Pageman over-representation analysis [55] was carried out for all three species and the significantly over-represented categories (Fisher’s test, *p* < 0.05; Table S3) in the up- and down-regulated genes across at least three treatments were identified (Fig. S1a, b). Many common categories were identified, revealing conserved functional responses at a transcriptome level to treatments (Fig. 2 a). As expected, genes encoding oxidative phosphorylation, tyrosine kinase-like protein kinases, ABC transporters and miscellaneous oxidoreductases were significantly enriched in the up-regulated gene-sets of all three species, as well as genes encoding APETALA2/ETHYLENE responsive element binding factors (AP2/ERF), heat shock factors (HSF) and NAC and WRKY domain containing TFs (Fig. 2 a). Enrichment of photosynthesis, RNA biosynthesis and RNA processing were observed among the down-regulated genes in all three species (Fig. 2b).

However, there were also notable differences between species. For example, protein modification was over-represented in up-regulated Arabidopsis genes in almost all sub-categories in response to all treatments, but this was seen less so in rice and barley, particularly for phosphorylation and tyrosine kinase-like protein kinases (Fig. 2a). The pattern of enrichment for protein biosynthesis functions in the down-regulated Arabidopsis genes also differed substantially compared to rice and barley (Fig. 2a). Thus, the effect of the treatments on protein modification, homeostasis and synthesis seemed to be different between dicots and monocots. Likewise, the processes of solute transport seemed to be less affected in Arabidopsis in down-regulated genes compared to the two monocots (Fig. 2b). The enrichment of vesicle trafficking among up-regulated genes was more similar between Arabidopsis and rice, while these were under-represented in barley. Overall, fewer functional categories were enriched in the up-regulated rice genes compared to Arabidopsis and barley, while the down-regulated genes had similar enriched categories. By contrast, the down-regulated genes in response to ABA were more similar in Arabidopsis and barley than rice e.g. for photosynthesis (Fig. 2b).

Species specific differences were also revealed in this way, for example the genes encoding oxidoreductases were enriched among the up-regulated genes following three stresses in Arabidopsis, five stresses in barley and only one stress in rice (Fig. 2b). Among the down-regulated genes, significant enrichment of genes encoding ribosomal proteins were observed following 3AT, ABA and MV treatment in both rice and barley, while this was only observed in response to ABA in Arabidopsis (Fig. 2b). Furthermore, the PLATZ family of TFs in Arabidopsis, MYB family in rice and C2H2-ZF family in barley were enriched in the up-regulated genes in response to three stresses in each species respectively while not showing the same enrichment pattern in the other two species (Fig. S1a; Table S3). Thus, the number and enriched functional categories differed between species and treatments indicating these differ irrespective of phylogenetic distance.

### Responses of orthologous genes between species

In order to directly compare transcriptomic responses between the three species, orthologous genes were identified using Orthofinder [56] to define sets of orthologous genes, termed orthogroups [24], which contain orthologs and paralogs in the three species (Table S4a). In this way, 9371 orthogroups were identified containing DEGs responsive to at least one treatment in at least one species (Table S4b). Interestingly, 49% of these contained DEGs responsive to at least one treatment in one species, while the orthologous genes in the other two species remained unchanging in expression. Closer examination of these revealed differing numbers of OGs containing DEGs responsive in only one species for each treatment (Fig. S2a; Table S4b). For example, the largest number of OGs containing DEGs responsive to 3AT, SA and UV in only one species was for rice, indicating that despite the presence of orthologous genes for these in barley and/or Arabidopsis, differential transcriptomic regulation occurs for these in rice (Fig. S2a; Table S4b). Similarly, the largest number of OGs containing DEGs responsive to MV in only one species was for barley, suggesting barley specific transcriptomic regulation of these genes occurs (Fig. S2a; Table S4b). Thus, while orthology indicates conservation at the gene level, transcriptomic responses for these can differ between species.

**Fig. 3:**
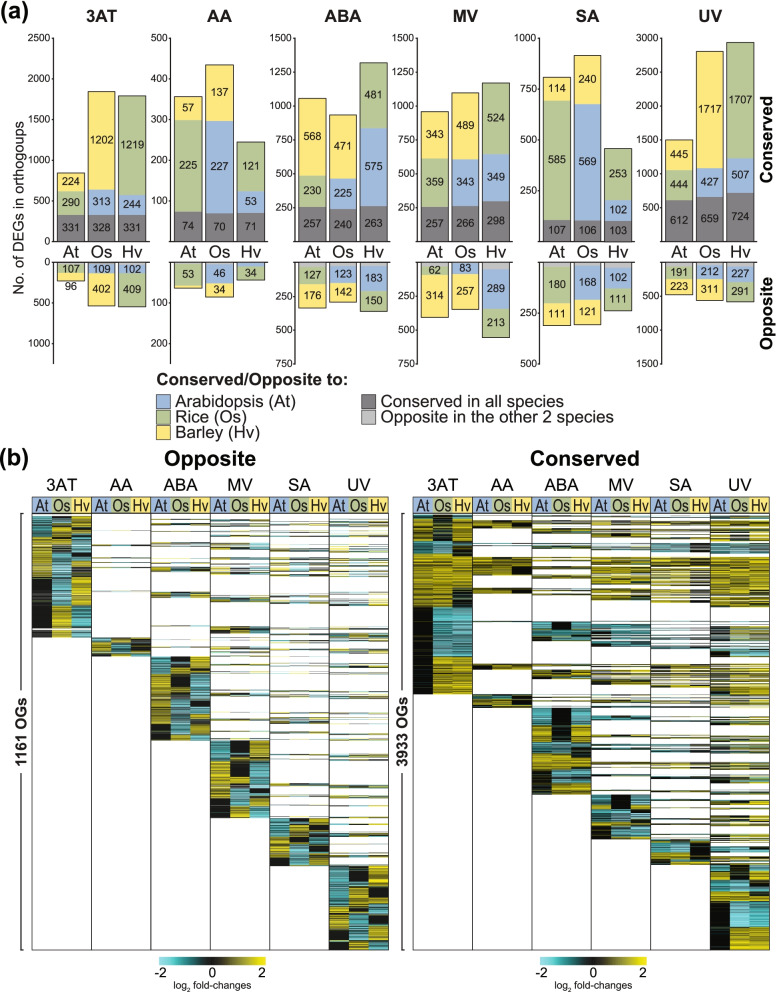
Transcriptome responses of orthologous genes in Arabidopsis, rice and barley following six different treatments. (a) The number of DEGs in Arabidopsis (At), rice (Os) and barley (Hv) that were orthologous and showed conserved or opposite responses to different stress treatments are indicated for each species. Conserved responses were defined as genes that were orthologous with at least one other species, that were also up- or down-regulated in transcript abundance in response to the treatment. An opposite response was defined as genes that were orthologous in at least one species, that showed the opposite response in one or both of the other species. Both conserved and opposite responses exclude any orthogroups in which genes displayed both up- and -down-regulation within the same orthogroup. Orthogroups were defined using Orthofinder as outlined in the methods section. (b) Heatmap of log2-transformed fold-changes for all orthogroups [24] that contain differentially expressed genes (DEGs) showing opposite and conserved responses (up/down-regulated) in all three species in response to the six treatments. 3AT = 3-amino-1,2,4-triazole; AA = antimycin A; ABA = abscisic acid; MV = methyl viologen; SA = salicylic acid; UV = ultraviolet radiation

Orthogroups containing DEGs responsive in the same manner (i.e. up/down-regulated) between species were defined as common, while those containing oppositely responsive orthologous DEGs between at least two species were defined as opposite (Fig. 3a; Table S4b). Of the 9371 OGs, 3933 OGs contained genes that showed common responses between at least two species in response to at least one treatment, while 1661 OGs contained genes that showed opposite responses (Table S4b). For orthogroups containing more than one gene, if the group contained some genes that were up-regulated and other genes that were down-regulated in response to a specific treatment from the same species within an orthogroup, those genes were excluded from the analyses for that treatment, for that species (Table S4b). In this way 821 multi-gene OGs were identified that contained both up-regulated and down-regulated genes in at least one species and 80 of these contained both up-regulated and down-regulated genes in at least two species (Table S4b).

Closer examination of OGs revealed that of the 2758, 7032 and 6107 DEGs in Arabidopsis, rice and barley respectively, responsive to 3AT (Fig. 1a), 845, 1843 and 1794 genes had orthologues that showed a common response with at least one other species (Fig. 3a). In Arabidopsis, the 845 DEGs represented 30% of all DEGs responsive to 3AT, with 331 of these genes showing a conserved response with their orthologous genes in both rice and barley, while 290 and 224 genes showed a conserved response with rice only and barley only, respectively (Fig. 3a). By contrast, of all Arabidopsis DEGs responsive to 3AT (Fig. 1a), 8% (228 genes) showed opposite responses to rice and/or barley. Twenty-five genes showed an opposite response to orthologous genes in both rice and barley, while 107 and 96 genes had an opposite response to orthologous genes in rice and barley only, respectively (Fig. 3a). The conserved responses and opposite responses to 3AT in barley and rice contained more than twice as many genes as Arabidopsis. Most of these, i.e. 1202 and 1219 genes, showed a conserved response between rice and barley, respectively, while 402 and 409 were oppositely responsive (Fig. 3a). Similar trends were observed in response to UV with 1501, 2803 and 2938 DEGs showing a conserved response with at least one other species (Fig. 3a), making up 27 to 39% of all DEGs (Fig. 1a).

Overall, at least twice as many genes showed conserved responses with one or more species than those that showed opposite responses (Fig. 3a). In barley more genes showed a conserved response with orthologous rice genes than Arabidopsis genes for 3AT, AA, MV, SA and UV (Fig. 3a). Similarly, in rice greater conservation was seen with the orthologous genes in barley in response to 3AT, ABA, MV and UV (Fig. 3a). Thus for at least four stresses, rice and barley showed more conservation in their response compared to Arabidopsis. However, greater conservation with Arabidopsis for rice in response to AA (227 genes) and SA (569 genes), and barley in response to ABA (575 genes) indicates that there are exceptions where greater similarity with Arabidopsis is seen (Fig. 3a).

Examination of the oppositely responsive genes revealed that in response to 3AT and UV, 7 to 9% of all genes (228–581 DEGs) showed opposite responses in at least one other species (Fig. 3a). Oppositely responsive genes in rice make up 10 and 11% of all DEGs responsive to ABA and MV, respectively, with 293 and 345 genes observed in these sets, while the 238 oppositely SA-responsive DEGs in barley make up 14% of all the DEGs (Fig. 3a). Thus, apart from AA, 228 to 581 DEGs were orthologous to oppositely responsive genes in at least one other species (Fig. 3a), making up 6 to 14% of all responsive DEGs in all five stresses. Thus, when only the DE orthologous genes are considered, there is greater conservation observed based on phylogeny (Fig. 3) than with the number of genes and enriched functional categories (Figs. 1 & 2). However, the greater conservation between Arabidopsis and rice for AA and SA, and Arabidopsis and barley for ABA (Fig. 3a) reveals diversification of responses do occur independent of phylogeny.

Overall, the examination of oppositely responsive DEGs revealed 1661 orthogroups containing 2275, 2365 and 2426 orthologous DEGs in Arabidopsis, rice and barley, respectively, that were oppositely responsive to at least one species and in at least one treatment (Fig. 3a). Remarkably, 38% of these 1661 orthogroups also showed opposite responses in more than one treatment, with 628 orthogroups containing 1024, 1083 and 1115 DEGs in Arabidopsis, rice and barley that were oppositely responsive in more than one stress (Fig. 3b). The greatest number of oppositely responsive genes within orthogroups were observed in response to UV, followed by MV, 3AT, ABA and SA with the smallest number observed for AA (Fig. 3b; Fig. S2b). When the numbers of specific and overlapping oppositely responsive OGs were examined, 407 out of the 1161 OGs showed overlapping responses i.e. also opposite in other stresses. For example, 200 OGs out of the 445 OGs within the oppositely responsive orthogroups in response to 3AT also showed opposite responses in at least one other stress (Fig. S2b).

Similar examination of genes showing conserved responses (Fig. 3a) revealed 3933 orthogroups containing 5186, 5560 and 5680 Arabidopsis, rice and barley orthologous DEGs, respectively, shared a conserved response with at least one other species and in at least one stress (Fig. 3b). Visualisation of these revealed that many of these show conserved responses in more than one stress, making up 48%, i.e. 1874 out of the 3933 orthogroups (Fig. 3b; Fig. S2b). When the numbers of treatment-specific and overlapping OGs were examined per stress, it is revealed that apart from UV, the majority of orthogroups showed overlapping responses in other stresses (Fig. S2b). This is consistent with previous findings that also revealed dynamic expression responses among orthologous genes (defined as Expressologs) across different plant species [57], highlighting the value of considering both expression and orthology when examining different species.

### Identification of common responsive genes across species

**Fig. 4:**
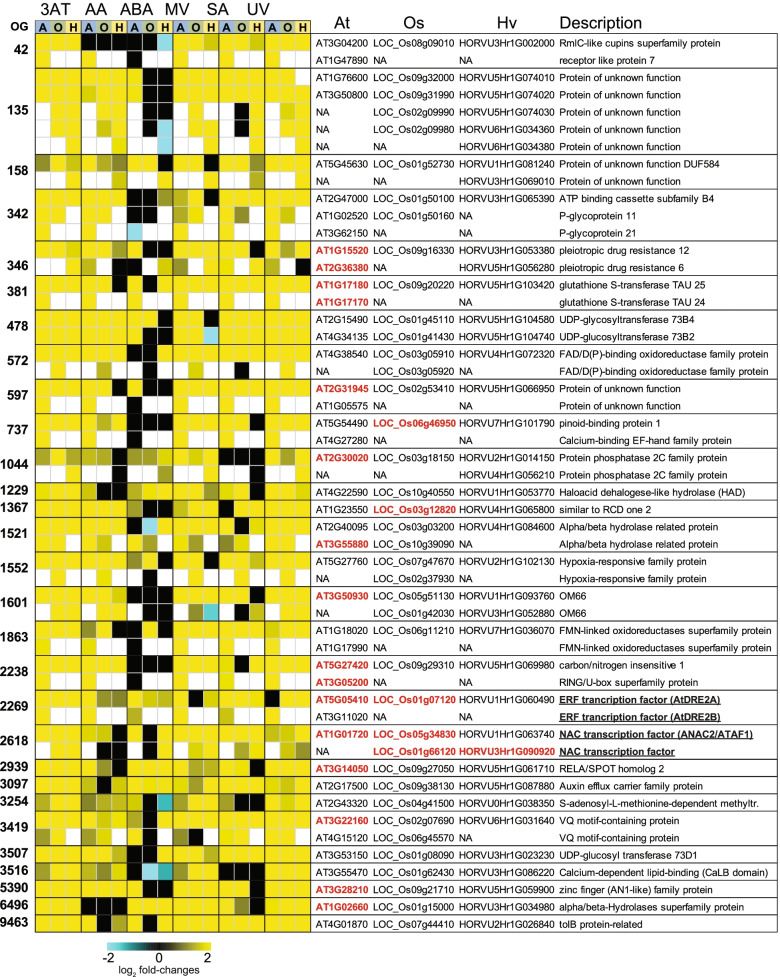
Conserved responses in gene expression of orthologous genes following treatments in Arabidopsis, rice and barley. Log2-transformed fold-changes are shown as a heatmap for the 20 orthogroups [24] that contained differentially expressed genes (DEGs) showing conserved responses across 4 out of 6 treatments (3AT = 3-amino-1,2,4-triazole, AA = antimycin A, ABA = abscisic acid, MV = methyl viologen, SA = salicylic acid, UV = ultraviolet radiation) for each of the three species (A = Arabidopsis, O = Rice and H = Barley). Genes with known functions (Supplemental Table 7a) (mutants/over-expressors) in development, defence and/or redox homeostasis are indicated in red. Underlining indicates genes encoding transcription factors

Identification of common responses of the DEGs in at least 3 out of 6 treatments across the three species revealed 105 OGs with 158, 146 and 157 Arabidopsis, rice and barley genes, respectively (Table S5a). GO annotations of the Arabidopsis genes in this set revealed 93 of the 158 genes were in the response to stimulus category and over-representation analysis revealed the top two categories were; response to molecule of bacterial origin and response to chitin (Fisher’s test, FDR *p* < 0.05) (Table S6a). Six of the top ten significantly enriched categories involved response to oxygen, decrease in oxygen levels or hypoxia (Table S6a). Orthologous DEGs that showed an up-regulated response in at least 4 out of 6 treatments in all three species revealed a set of 29 OGs with 43, 40 and 39 genes in Arabidopsis, rice and barley respectively (Fig. 4; Table S5b). This set identifies genes with both genetic and transcriptomic conservation, suggesting that the regulation of these may also be conserved.

Closer examination of the Arabidopsis genes revealed that 24 genes showed high or maximal expression during senescence in leaves compared to all other developmental tissues in Arabidopsis [58, 59] (Table S7a). It was also observed that ten genes out of the 43 genes (23%) produce known cell-to-cell mobile mRNAs, representing a significant enrichment (chi-square, p < 0.05), considering only 2006 genes (approx. 6.7% of all genes in the genome) in total are cell-to-cell mobile [60]. Examination of gene functions revealed 19 out of 43 Arabidopsis genes in this common set are known to have a functional role, whereby alteration e.g. mutation/ RNAi/ over-expression of these genes resulted in plants with altered stress responses/development (Table S7a). Five of these genes resulted in altered redox-related changes in the cell (Table S7a). For example mutation of the gene encoding GLUTATHIONE S-TRANSFERASE (CLASS TAU) 24 (GSTU24, At1g17170) caused increased overall GST activity and altered redox homeostasis [61]. A gene encoding a transmembrane protein (At2g31945) and another encoding a glycerolipid A1 lipase annotated as PLASTID LIPASE 2 (At1g02660) (Fig. 4) also resulted in reduced oxidative stress tolerance in knock-out plants for these genes [62]. Similarly, the zinc finger (AN1-like) family protein STRESS-ASSOCIATED PROTEIN [48] 12 has been shown to be under redox-dependent regulation [63] and elevated expression of its regulator miR408 leads to SAP12 induction as well as an increase in cellular antioxidant capacity [64] (Table S7a).

Additionally, alterations in the expression of seven genes common in the three species (Fig. 4) results in altered biotic stress responses (Table S7a), including for the two ABCG genes (ABCG40 and ABCG34). Mutations of these two results in compromised *Phytophthora brassicae* and *Phytophthora capsici* as well as necrotrophic pathogen resistance, respectively [65, 66]. Similarly, the loss-of-function of a MAPK phosphatase (At2g30020) [67], the over-expression of a cytochrome BC1 synthesis-like outer mitochondrial membrane protein OM66 (At3g50930) [68] and the over-expression of ATL6 (At3g05200) and ATL31 (At5g27420) [69, 70] result in increased resistance to *Pseudomonas syringae* infection, while the silencing of the VQ motif-containing gene JAV1 (At3g22160) enhances jasmonate-regulated defence against *Botrytis cinerea* [71] (Table S7a). Two genes encoding auxin transporting ATP-BINDING CASSETTE proteins (At2g47000 and At3g62150) as well as a calcium binding protein encoding gene (At4g27280) were observed to result in altered auxin responses and root formation in corresponding mutant plants [72–74]. Thus, this set of stress-responsive genes are conserved across Arabidopsis, rice and barley and are involved in redox and defence maintenance, and their conservation in expression implies that these roles and possibly their regulation may also be conserved in the three species. In fact, loss-of-function of the calcium-binding protein OsCCD1 (LOC_Os06g46950), an orthologue of At4g27280 (OG0000737) (Fig. 4), is less tolerant to osmotic and salt stresses while overexpression significantly enhances this tolerance in rice [75] (Table S7a).

The orthogroup OG0002618 represents an example of conservation across all three species and contains NAC TFs that are important for stress responses (Fig. 4) [76]. ATAF1 (ANAC2; AT1G01720) plays an important role in the crosstalk between abiotic and biotic stress response pathways and acts as an ABA-dependent regulator of defence and plant stress tolerance [77, 78]. Overexpressing ATAF1 in Arabidopsis increases drought tolerance [79] and overexpression of this Arabidopsis gene in transgenic rice conferred tolerance to salt stress [78]. Overexpression of the closest ATAF1 homologs in transgenic rice confers cold and salt tolerance (OsSNAC2; LOC_Os01g66120) [80] as well as drought tolerance (OsNAC52; LOC_Os05g34830) [81] in an ABA-dependent matter. An orthologue of this in barley, HvNAC6 (HORVU3Hr1G090920), mediates ABA-dependent defence responses and corresponding knock-down lines are more susceptible to powdery mildew [82] (Table S7a).

### The mitochondrial dysfunction stimulon is a conserved organellar response

Mitochondria play important roles in stress responses, both by providing energy and working as stress sensors and signalling hubs [83]. The mitochondrial dysfunction stimulon (MDS) is part of the mitochondrial retrograde signalling pathway that signals mitochondrial dysfunction caused by genetic, pharmological or environmental conditions to alter the expression of nuclear genes such as the mitochondrial stress marker alternative oxidase AOX [84]. NAC TFs such as ANAC013 and ANAC017 bind a *cis*-regulatory motif, called mitochondrial dysfunction motif (MDM) which is present in the promoter of several genes that have altered expression in response impaired mitochondrial function [84]. A motif search identified these binding sites to be present in the promotor region of various *AOX* genes in rice, barley and wheat which suggests conserved MRR pathways across plant families. Conservation of these pathways in monocots is further supported by interaction of ER-membrane bound OsNAC054, involved in ABA-induced leaf senescence in rice that has been shown to specifically bind the MDM [85].

**Fig. 5:**
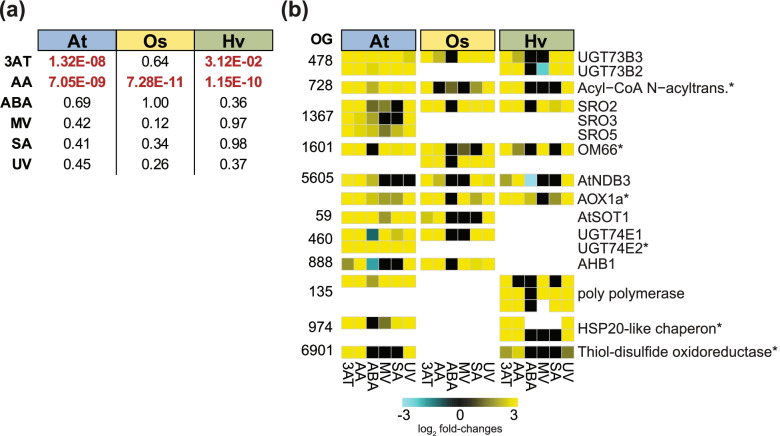
Mitochondrial dysfunction motif (MDM) enrichment in stress treatment-specific gene sets and conservation of potential mitochondrial dysfunction stimulon (MDS) genes in Arabidopsis (At), rice (Os) and barley (Hv). (a) Enrichment analysis of the MDM in the upstream promotor region of specific subsets containing all differentially expressed genes (DEGs; |log2 (fold change)| > 1, FDR < 0.05) for each stress treatment in At, Os and Hv; 3AT = 3-amino-1,2,4-triazole, AA = antimycin A, ABA = abscisic acid, MV = methyl viologen, SA = salicylic acid, UV = ultraviolet radiation. The presence of the stringent MDM in all expressed genes in each species and each stress was analysed for significance (*p*-values; chi-square test, significance (*p *<0.05) is indicated in red font). (b) Heatmap showing gene expression levels for DEGs of MDS candidate genes in At, Os and Hv. All genes contained the MDM in the 2 kb promotor region and showed differential expression in response to AA and/or 3AT and at least 3 out of 6 stresses. The number of each orthogroup (OG) is indicated and known MDS genes are highlighted with an asterisk. Gene names/annotations refer to the genes in Arabidopsis

Our orthology analysis showed conservation of genes encoding mitochondrial proteins, across Arabidopsis, rice and barley (Table S4 & S5), with common up-regulation of *OM66* in four out of the six treatments (Fig. 4). In order to investigate this conservation in monocots and dicots, a pattern search of the stringent MDM (CTTGNNNNNCAMG) was conducted in the promotor region (2 kb upstream of the translation start site) for all genes that were expressed in this study, not allowing for any permutations, using the Regulatory Sequence Analysis Tools [54]. A motif enrichment analysis (*chi*-square test, *p* < 0.05) with treatment specific subsets was performed (Table S8a). For AA responsive DEGs, the motif is significantly enriched across all three species indicating conserved pathways in response to mitochondrial dysfunction and MRR (Fig. 5, Table S8). By contrast, the MDM in the promoters of 3AT-responsive DEGs is only conserved in Arabidopsis (*p* value < 0.001; chi-square test) and barley (p value < 0.05; chi-square test), but not in rice (p value = 0.64). ABA, MV, SA and UV show no MDM enrichment for all three species revealing that the gene induction via the MDM is likely specific to mitochondrial oxidative stress, in agreement with functional studies in Arabidopsis [84, 86].

To identify new MDS candidate genes, the gene regulatory network of ANAC017 [25] was analysed for the presence of the MDM (Table S8b). ANAC017 is a regulator in the MRR in Arabidopsis and a direct positive regulator of *AOX1a* [86]. It directly interacts with the MDM and the promotor of ANAC013, an MDS gene itself [84]. All AtNAC017 gene regulatory network genes containing the MDM that were differentially expressed following AA and 3AT treatment and responsive in at least one other treatment (3 out of 6 treatments) were filtered, in accordance to the MDM enrichment analysis. The final list of 82 candidate genes were hierarchically clustered together with the MDS genes (Fig. S3). The list of candidate genes in Arabidopsis contains 7 UDP-glycosyltransferases (UGTs), including known MDS gene *UGT74E2* (At1g05680), which plays a role in organelle signalling [87] (Fig. S3). The induction of UGT genes under abiotic and biotic stress indicates that they may be involved in modifying a variety of hormonal signalling pathways under mitochondrial retrograde signalling [88]. The presence of a biotic stress responsive cell wall associated kinase [89, 90], links mitochondrial signalling to cell wall, as previously shown for ANAC017 that could restore cell wall growth in the presence of inhibitors to cellulose synthase in an unknown manner [91]. Other ANACs in the candidate list are ANAC044 (At3g01600), characterised to mediate stress induced cell cycle arrest [92], ANAC053 (At3g10500) which mediates proteasome stress [93] and ROS production during drought [94], and ANAC055 (At3g15500) with a role in mediating drought responses [95]. Additionally, three members of the SIMILAR TO RCD-ONE family (SRO2/3/5) are also in the list (Fig. S3). Combined these examples position mitochondrial signalling as an important hub for a variety of cellular signalling pathways, consistent with emerging roles for mitochondria in flooding response [25], interaction with touch signalling [96], and the role of ANAC017, considered the master regulator of mitochondrial retrograde signalling, in regulating growth, senescence and cell wall growth [25].

Based on this expanded list of candidates and known MDS genes, the conservation of MDS across the three species was analysed using the inferred OGs (Table S8c). Several OGs were identified that included MDM containing genes that also show similar expression patterns across the three species (Fig. 5b). These included AOX, which was used as a reference, *SRO2*, 3 and *5*, the alternative dehydrogenase *NDB3*, as well as several glycosyl transferases and Acyl-CoA N-acyltransferase that are present in all three species. Note, ABA does not appear to be a major regulator of the MDM-dependent response in rice and barley compared to Arabidopsis (Fig. 5b). In rice, none of these OGs respond to ABA, while in barley only *NDB3* is negatively regulated by ABA, and only *AOX1a* is slightly up-regulated. This may reflect a divergence in signalling pathways, as has been previously reported for biotic and drought stress [97]. The MRR marker gene *AtAOX1a* as well as *AOX1a* in rice and barley have previously been reported to contain the MDM, which was confirmed in this analysis. AOX1a was up-regulated in at least 5 out of 6 stress treatments, although in barley *AOX1a* is not responsive to MV (or any other *AOX* gene). Other MDS genes like At*OM66* and Acetyl-CoA N-acyltransferase (At2G32020) are conserved as well and have rice and barley orthologues that also have the MDM and similar expression responses (Fig. 5b). Novel MDS candidates identified here are At*SRO2/3/5* and its orthologues in barley and rice. These are members of the plant specific SRO gene family that play important functions in stress responses and development. This family includes RADICAL-INDUCED CELL DEATH 1 (RCD1), a nuclear-localized transcriptional regulator, which was recently shown to suppress the activity of ANAC013 and ANAC017 and increased expression of MDS genes affecting ROS homeostasis in the chloroplasts [46]. The corresponding OG with these MDS candidates contains OsSRO1c, which is a known mediator of multiple abiotic stresses [45], and was responsive in all treatments in this study, except ABA (Fig. 5b). OsSRO1c is a direct target gene of OsSNAC1 and plays a role in drought and oxidative stress tolerance [98]. The orthologue in barley shows the same expression pattern as Os*SRO1c* and represents an interesting target as there are no studies to date that have analysed the *SRO* gene family in barley. Several other OGs containing new candidate MDS genes show a conserved response across species, including *NDB3*, which along with *NDB2* [99, 100] may act with *AOX1a* to form a conserved and complete respiratory chain under stress conditions in all three species [48].

### Oppositely responsive orthologous genes indicates transcriptomic diversity between species

**Fig. 6:**
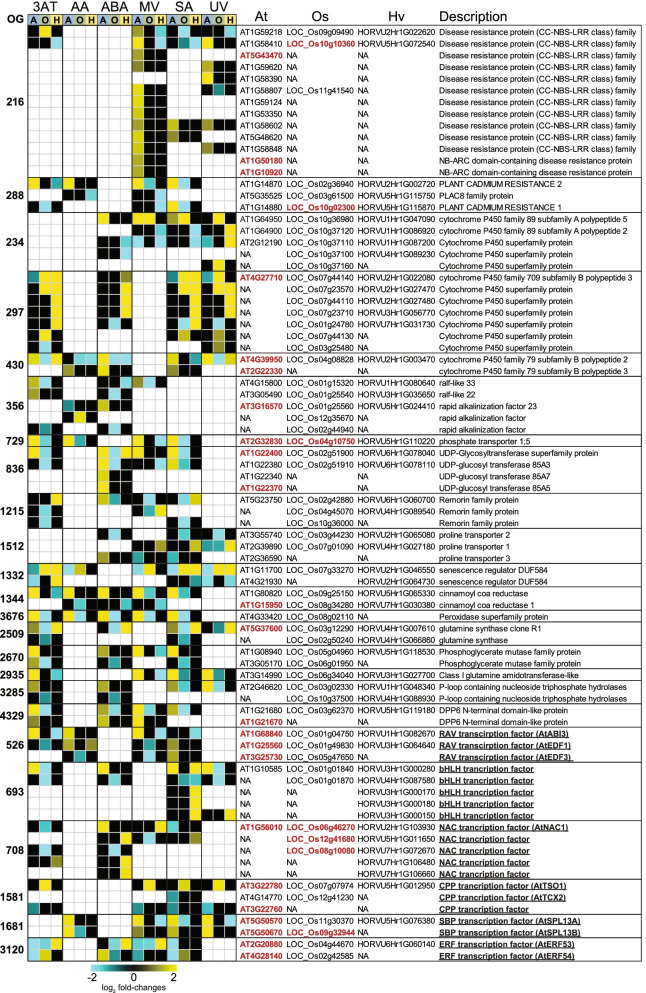
Oppositely responsive orthologous genes following treatments in Arabidopsis, rice and barley. Log2-transformed fold-changes (FDR < 0.05) are shown as a heatmap for the 24 orthogroups [24] that contained differentially expressed genes (DEGs) showing opposite responses (up/down-regulated) in 4 out of 6 treatments (3AT = 3-amino-1,2,4-triazole, AA = antimycin A, ABA = abscisic acid, MV = methyl viologen, SA = salicylic acid, UV = ultraviolet radiation) for each of the three species (A = Arabidopsis, O = Rice and H = Barley). Arabidopsis genes with known functions (ST 7b) in development, defence and/or redox homeostasis are indicated in red. Underlining indicates genes encoding transcription factors

Of the 1661 OGs containing oppositely responsive genes, 102 orthogroups were oppositely responsive between species in at least three stresses. Of these, 24 OGs containing 58, 58 and 51 genes in Arabidopsis, rice and barley, respectively, were oppositely responsive between two species in at least four stresses (Fig. 6; Table S9). When the Arabidopsis genes in these two sets of genes were examined for over-represented GO biological processes (Fisher’s test, *p* < 0.05), the top categories included intracellular signal transduction and response to hormone stimulus in both sets (Table S6b). Notably, nine of the 58 Arabidopsis genes, (15% vs. 6.7% in the genome) also produced known cell-to-cell mobile mRNAs representing an enrichment (chi-square, p < 0.05) [60] (Fig. 6). Examination of the functions of the oppositely responsive gene-sets (Fig. 6) revealed known roles for many of these genes (indicated in red font) in biotic and abiotic stress in Arabidopsis and rice (Fig. 6; Table S7).

A large orthogroup containing 13 out of the 58 Arabidopsis genes (opposite in 4 treatments) encoding disease resistance genes were induced in Arabidopsis following MV, SA and UV treatment, while their orthologous genes were down-regulated in rice and/or barley (Fig. 6). Two of these were NB-ARC domain containing disease resistance genes, annotated as CEL-ACTIVATED RESISTANCE 1 (CAR1; At1g50180) and LOCUS ORCHESTRATING VICTORIN EFFECTS1 (LOV1; At1g10920) (Fig. 6), which have key roles in defense in Arabidopsis (Table S7b). CAR1 is a key host immune receptor, responsible for recognising *P.syringae* effectors [101], while LOV1 elicits a resistance-like response that results in disease susceptibility [102]. The loss-of-function of CAR1 and LOV1 resulted in increased susceptibility to *P.syringae* and *Cochliobolus victoriae*, respectively in Arabidopsis [101, 102]. Interestingly, the overexpression and suppression of the rice protein LOW SEED SETTING RELATED (OsLSR; LOC_Os10g10360) in the same orthogroup (OG0000216) leads to a constitutively activated immune system [103]. Similarly, the over-expression of another disease resistance protein (At5g43470) resulted in increased resistance to the cucumber mosaic virus [104], while the loss-of-function of two cytochrome p450 family proteins (At4g39950 and At2g22330) and a UDP-Glycosyltransferase superfamily protein (At1g22400) resulted in increased susceptibility to *Alternaria brassicicola* and *P.syringae*, respectively [105, 106]. Thus, altering the expression levels of these appear to functionally alter biotic stress responses. Notably, all of the aforementioned genes were up-regulated in Arabidopsis, while their orthologous genes in rice and/or barley were down-regulated (Fig. 6).

Genes in this set were also functional in abiotic stress responses (Fig. 6). For example, OG0002509 contains two glutamine synthetases in rice that are down-regulated in response to ABA, MV, SA and UV while the Arabidopsis and barley orthologues in the corresponding group are either up-regulated or unresponsive (Fig. 6). Concurrent overexpression of OsGS1;1 and OsGS2 has been shown to enhance osmotic, salinity and MV induced photo-oxidative stress tolerance [107]. Similarly, the loss of AtGLN1;1 (At5g37600) leads to impairment of redox homeostasis in chloroplasts of MV-treated leaves [108]. In addition, the combinatorial loss of ATGLN1;1 and other glutamine synthetases impacts the capacity to tolerate abiotic stresses in Arabidopsis [108]. In contrast to these up-regulated Arabidopsis genes, the gene encoding the cytochrome p450 superfamily protein CYP709B (OG0000297) was down-regulated or unresponsive in Arabidopsis, while its orthologous genes in barley were induced in response to 3AT, SA and UV (Fig. 6). Loss-of-function of this gene in Arabidopsis resulted in increased sensitivity to ABA and salt stress [109]. Similarly, we found AtRAV2/ABI3 (At1g68840) was induced under 3AT, AA and MV, while its rice and barley orthologues were down-regulated (Fig. 6). Loss-of-function of AtRAV2 also alters the salt stress responses with *rav2* mutants showing improved seed germination under salt conditions [110]. Interestingly, AtRAV2 expression is reduced under salt stress [110], while one of its rice orthologues (OsRAV2; LOC_Os05g47650) is induced under salt stress [111] and has also been implicated as having a functional role in the rice salt stress response [112]. Similarly, a UDP-glycosyl transferase 85A5 encoding gene in Arabidopsis (At1g22370) is significantly induced in response to 3AT, ABA and SA, while its rice orthologues are down-regulated and its barley orthologue only up-regulated under ABA treatment (Fig. 6). This gene is known to be induced under salt stress in Arabidopsis, with ectopic expression in tobacco resulting in improved salt tolerance in transgenic plants [113]. Thus, alteration in the expression of genes in this subset appears to directly affect abiotic stress responses, with diversity in expression seen for genes with known roles in salt stress tolerance.

Lastly, contrasting expression was observed between Arabidopsis, rice and barley for the genes encoding CINNAMOYL COA REDUCTASE (CCR) 1 and CCR 2 in Arabidopsis (AT1G15950 and AT1G80820) and while *CCR1* showed no change in expression, *CCR2* was induced in Arabidopsis under five of the treatments (apart from UV) while their rice and barley orthologues were reduced in expression (Fig. 6). CCRs have an important role in lignin biosynthesis and when CCR1 was knocked-out in Arabidopsis, stunted growth and delayed development was observed while CCR1 knockouts in *Medicago truncatula* showed more significant impairment resulting in most plants not surviving [114]. Thus, despite orthology, the expression and function of these genes may differ between Arabidopsis, rice and barley indicating a disparity between orthology and expression that must be considered for translational work between species, both in terms of the effects on development and under stress, such as salt stress.

### Diversity in transcription factor expression despite orthology

**Fig. 7:**
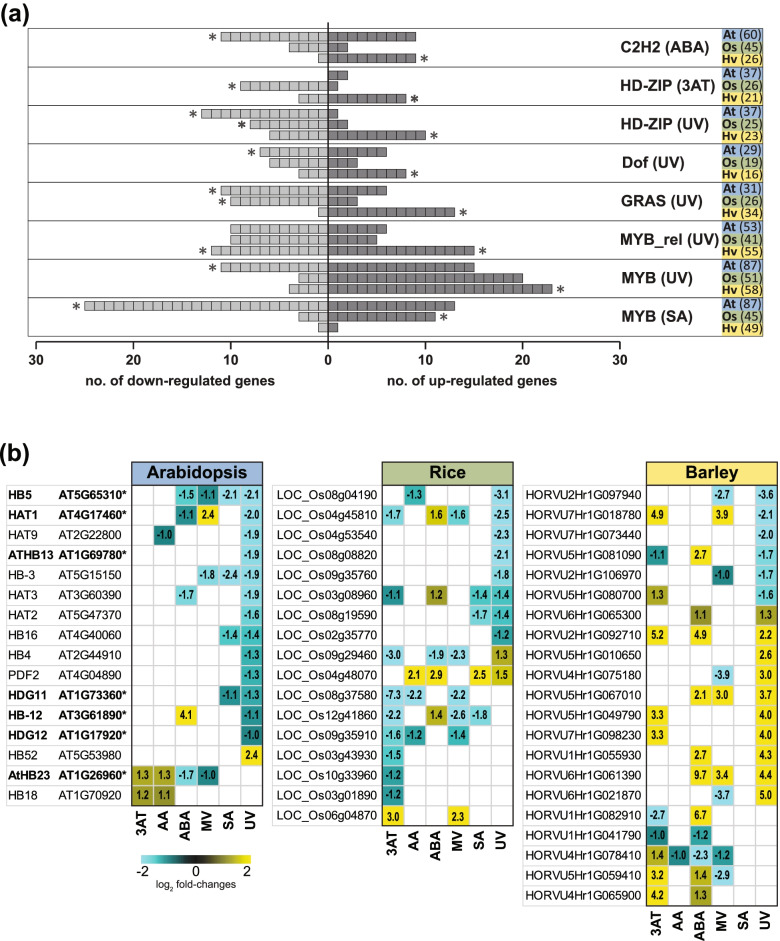
Oppositely responsive transcription factor families. (a) The number and expression of transcription factors in each family that were significantly enriched (*p* < 0.05; indicated by an asterisk) in oppositely responsive gene-sets (up/down-regulated) in Arabidopsis, barley and/or rice. (b) The expression of all homeodomain/homeobox-leucine zipper encoding proteins that were differentially expressed in response to 3AT and/or UV treatment in in all three species. Note Arabidopsis genes annotated with an asterisk indicate genes that have an experimentally confirmed function in development, particularly root and trichome branching (ST 7c)

All genes encoding TFs from the three species [115] were analysed for enrichment of specific families within the DEG sets. ERF and NAC TFs were enriched (hypergeometric distribution; *p*-value< 0.05) in the DEG sets in at least two species across all six stresses (Fig. S4, S5a; Table S10). The greatest number of ERFs (Arabidopsis: 32, rice: 22, barley: 26) and NAC TFs (Arabidopsis: 31, rice: 27, barley: 46) induced across all three species was in response to 3AT and UV (Fig. S5a). Similarly, WRKY, bHLH, HSF, MYB and C2H2 TFs were significantly enriched (*p* < 0.05; Fig. S5b; Table S11) in at least two species in response to five of the six treatments. Of these, WRKY factors were enriched in the up-regulated gene sets, similar to ERFs and NACs (Fig. S5b). By contrast, bHLH TFs showed conserved enrichment among down-regulated genes in Arabidopsis and barley in response to ABA, MV and SA, while MYB encoding genes were enriched among the down-regulated genes only in Arabidopsis in response to ABA and MV (Fig. S5b). Given these species-specific differences, we further examined the TF families to identify families that were enriched in oppositely responsive DEGs between species. Five TF families (C2H2, HD-ZIP, Dof, GRAS and MYB) were identified that were enriched in the up-regulated gene sets of one species and the down-regulated gene set (or vice versa) of another species in response to the same stress (Fig. 7a; Table S12). Notably, all five families were enriched in the up-regulated gene sets in barley, while an enrichment of the same family was observed in the down-regulated gene set(s) in Arabidopsis and/or rice (Fig. 7a). For example, in response to ABA, the C2H2 family was enriched among the down-regulated genes in Arabidopsis while this family was enriched in the up-regulated genes in barley (Fig. 7a).

As TFs can complement other TFs in the same family, we examined the expression of all DEGs in each of the five families enriched in the oppositely responsive sets (Fig. 7a). This revealed that despite being enriched in oppositely responsive subsets, there are members in the TF families that are up-regulated and down-regulated within each family, making it difficult to ascertain whether the opposite responses represent functional differences. Nevertheless, this examination identified families in which the majority of genes show opposite transcriptomic responses (Fig. 7a) and for many of these their responses were conserved across more than one stress (Fig. S6). For example, in response to SA and UV, the majority of MYB factors are up-regulated in rice and barley, while most are down-regulated in Arabidopsis and several of these shows the same up/down-regulated response across both stresses in each species (Fig. S6). Similarly, all but one GRAS family TFs are up-regulated in barley in response to UV and similarly to 3AT, while the majority are down-regulated in Arabidopsis and many of these maintain this down-regulation in response to MV (Fig. S6). This supports the idea of a conserved stress-responsive regulation of these TFs within a single species.

For the homeodomain (HD-ZIPs) family, the opposite pattern of regulation between species is observed in response to both 3AT and UV (Fig. 7a&b) with HD-ZIPs enriched in the up-regulated genes in barley, down-regulated genes in Arabidopsis (UV only) and down-regulated genes in rice (3AT and UV) (Fig. 7a). Only one HD-ZIP factor in Arabidopsis and two in rice were up-regulated while the most of these were highly induced in response to UV in barley (Fig. 7b). In Arabidopsis, of the 14 DEGs encoding HD-ZIPs in response to UV, 13 were down-regulated and six have been experimentally shown to have functionally significant roles in development and/or stress, with a loss-of-function resulting in altered function [116–118]. Interestingly, the expression of this family in barley revealed 10 of the 16 DEGs encoding HD-ZIPs were up-regulated and five of these by > 4-fold (Fig. 7b).

## Discussion

Comparison of the transcriptome responses of a model species (Arabidopsis) and two crop species (rice and barley) to six treatments designed to mimic stress responses (Table 1) revealed much needed insight into the level of genetic and transcriptomic conservation between these species. The examination of the responses to hormones (ABA and SA); treatments that cause oxidative stress (3AT, MV); inhibit respiration (AA) or induce genetic damage [53] in parallel for these three species unveiled similarities and differences in the abiotic stress responsive pathways that are affected (Table 1) revealing a depth of knowledge that is not possible with single treatment/species studies. The use of marker genes ensured the efficacy of the treatment, albeit for two treatments in barley a later time point was used (6 h rather than 3 h), to obtain similar responses as in rice and Arabidopsis. When carrying out cross species comparisons the need to align as many parameters as possible is necessary to validate the comparisons. Given the extensive optimisation of the treatment and time in Arabidopsis previously this was kept where possible, as many functional studied have been carried out based on the transcript changes to validate their role in stress response. Likewise for the tissue, early development stage was used for all species, with two-week-old Arabidopsis [119] (growth stage: 1.06), rice and barley [120] (both growth stage 13). While there are minor differences in development between Arabidopsis 1.06 and rice and barley stage 13 (equivalent to Arabidopsis 1.03), the differences in development are small (see below). Overall, the validity of the comparisons is evident by; i) 80% of orthologues show similar responses between species, ii) conserved responses are four times more abundant than non-conserved responses, and iii) when opposite responses were observed this could not be accounted for by the small, growth stage difference as the genes that were responding in an opposite pattern displayed a correlation of r = 0.97 between day 7 and 21 of Arabidopsis (Table S9).

This approach revealed commonalities and differences in responses that will provide a roadmap for translation research, helping with the transfer of knowledge gained in a model system to crop species. This study revealed that the responses to treatments (Table 1) were diverse in number, gene ontology, orthology and expression. Analyses of the number of transcripts that responded to the treatments in the different species did indicate a trend in terms of specificity or conservation (Fig. 1). Notably, the overlap in response to the various treatments was greater in Arabidopsis than it was for barley and rice, and this may indicate that the regulatory hierarchy in Arabidopsis is more shared than it is in other species (Fig. 1).

While these differences can now be tested experimentally, it interesting that the changes in TFs expression in many families differed fundamentally (Fig. 7). A recent review of TFs across algae, non-seed plants and seed plants revealed, with examples therein, both a link between conservation in TF expression and conserved function, and conversely examples of TFs with functions that were not deeply conserved as having distinct or lineage specific expression patterns [121]. Thus, the extensive interaction of signalling networks involving TFs that have emerged from studies in Arabidopsis may be more limited or different in other species. For example, the two Arabidopsis orthologues to OsMYBS1, which has a role in sugar and hormone mediated signalling in rice were found to play opposite roles to each-other in regulating glucose and ABA signalling in Arabidopsis, indicating distinct regulatory roles of some TFs in these species [122]. These potential differences have significant implications for the translation into crop species, as under field conditions, plants are also exposed to multiple non-optimal conditions [123, 124]. While the response to these multiple conditions may be highly integrated in one species, it is possible they may trigger multiple or even antagonistic parallel pathways in other species. Notably, hormone pathways were enriched among the oppositely responsive sets identified in this study. Given the interaction of anterograde, retrograde and hormone signalling pathways [123, 125, 126], it will be essential to understand these differences for translational research.

One difference observed in the response to the treatments were GOs related to protein homeostasis that differed significantly between Arabidopsis, rice and barley. The rate of growth in Arabidopsis is inversely related to protein turnover [127], and protein synthesis is energetically expensive [128]. Thus, the rate of protein turnover and synthesis is a major energy sink in plants, but also indicative of responses to stimuli. Differences in the treatment responses for protein homeostasis and vesicle trafficking in Arabidopsis (Fig. 2a) may point to differences in these processes between plants and likewise for protein biosynthesis. Thus, both may indicate differences in protein turnover rates under adverse conditions between the three species, with possible consequences for growth and development. This facet needs to be further investigated if the principles of energy consumption limiting growth under adverse conditions are to be applied from Arabidopsis to other plants [129]. It should be noted that in this study the Arabidopsis accession Columbia was used and given differences between in the rate of protein turnover have been seen in Arabidopsis accessions [127], this may not necessarily be a reflection of a ‘typical’ response to stress. Another possible contributing factor is the lifespan differences in the three species, which impact mRNA and protein turnover.

The approach used in this study to define opposite responses was very conservative, in that it was restricted to orthology and all genes in the orthogroups needed to display the same trend. Therefore, the opposite responses outlined in this study are likely to be an underestimation of the differences between species. This can be seen to some degree when the expression of whole TF families is analysed (Fig. 7). The large differences in the number of genes from each family that were up- or down- regulated in abundance in response to the treatment indicates that there were fundamental differences in how genes were regulated. Thus, the common practise of expressing a TF from one species in another species to transfer a trait such as tolerance to a limiting condition may not result in all the desired effects. For example, OsAP2 and OsWRKY24 have been proposed to have opposite roles in rice and Arabidopsis, with these known to be positive regulators involved in increased lamina inclination, grain size and cell elongation in rice, while their overexpression in Arabidopsis resulted in reduced plant size and cell size [130]. Similarly, the outcome following over-expression of AtFD and AtFDP in transgenic rice was not as expected compared to that seen in Arabidopsis [131]. Thus, despite orthology, function and expression can clearly differ between species with differences in response of TFs to treatments observed in closely related monocot species, e.g. for GRAS and HD-ZIP TFs in response to UV. The differences in responses of TF families to stresses may indicate that while the upstream regulatory network may be different, the resulting responses of conserved target genes are similar. This may limit the utility of promoter motif searching cross-species which is widely used in research on crop species.

Despite similarities in promoter regions and significant orthology between genes, it is possible to observe opposite expression responses such as seen for phototropin genes in Arabidopsis and Brachypodium [132]. Similarly, despite orthology and experimental confirmation of identical function of the PAO/phyllobilin pathway in barley and Arabidopsis, the downstream effects of these proteins differed between the species [133]. Another example of these distinctions can be seen for rice PHYTOCHROME-INTERACTING FACTOR-LIKE1 (OsPIL1), which negatively regulates leaf senescence in rice with *ospil1* mutants senescing earlier than WT, while the opposite was observed for the orthologues of OsPIL1 in Arabidopsis *atpif4* and *atpif5* mutants [134]. Opposite effects were also seen for the effect of potassium deprivation on jasmonic acid related gene expression and downstream resistance to herbivorous insects, which again differed between barley and Arabidopsis, despite orthology of JA responsive genes [135]. These just represent some of the examples of where despite orthology, gene function and phenotypic effects differed between Arabidopsis, rice and barley, highlighting the need for resources such as this study, and other similar studies [57] to identify common and distinct responses between species, particularly those across multiple treatments, providing valuable information for further experimental design.

While there were notable differences in the observed species responses in this study, there were also notable similarities. The conserved responses observed between all three species to three or more stimuli revealed that fundamental response pathways have been conserved from perception to response at a gene level (Fig. 4). A good example of this was seen with the Arabidopsis mitochondrial dysfunctional response [84]. In response to AA, and to a lesser degree 3AT, the *cis*-regulatory motif in the promoter region of the DEGs is enriched in all three species (Fig. 5a). Furthermore, there are more MDS candidate genes present in Arabidopsis than previously defined. Thus, the mitochondrial regulatory pathway that is controlled by activation of latent ER bound NAC TFs and repressed by RCD like proteins is a common theme observed across monocots and dicots. In Arabidopsis, the role of the ANAC017 TF has now expanded as being involved in flooding responses, ageing and senescence and as a growth regulator [136]. Plants defective in ANAC017 grew and developed more rapidly than wild type controls with as much as 50% additional biomass accumulation, while plant with over-expressed ANAC017 displayed growth retardations [136]. However, detrimental effects were only observed with higher levels of over-expression. Thus, given the central role of this TF in stress responses by integrating mitochondrial and chloroplast energy metabolism with environmental conditions, and the conserved nature of this pathway across species, our study exemplifies how natural variation or modification of target genes may be used for agronomic purposes.

## Conclusions

The cross-species comparison of transcriptomic responses to the six treatments in this study showed overlap between stresses, more so for Arabidopsis than barley and rice. Over 5000 genes were both orthologous and showing conserved responses in at least two species in response to at least one treatment, indicating conservation across species of the relevant responsive pathways including stress responsive TFs such as ERFs, NAC and WRKY TFs. Closer examination of conserved genes showing common responses revealed several genes with known functions in stress response pathways including NAC [137] and ERFs TFs [138] as well as others (red font; Fig. 4). While the large overlap in the presence of orthologous genes showing conserved responses was to be expected, it was remarkable that 15–34% of orthologous DEGs between species show opposite transcriptomic responses. Examination of these identified genes with known roles in the biotic and abiotic stress response pathways, with known functions for some of these, whereby alteration in expression resulted in altered phenotypes in Arabidopsis (red font; Fig. 6). Thus, despite these genes being orthologous in rice and barley as well, it is possible that the shortlist identified here represents only a subset of genes with divergent regulation, particularly given that the opposite responses were observed following at least four independent treatments. The presence of six orthogroups containing oppositely responsive TFs out of the 20 that were opposite in at least four treatments indicates diversity in TF gene expression, further supporting the possibility of differential regulation between species. While this study compared the responses of Arabidopsis, barley and rice to various stimuli, only a single stimulus was used at a time and emerging studies highlight the importance of combination of stresses [139]. The differences observed in the overlapping responses to treatments and opposite responses means that more differences may emerge between species when combined stresses are applied.

## Methods

### Plant material and growth conditions

Rice seeds (Oryza sativa L. ssp. Japonica, cultivar Millin) were surface sterilized and germinated on a petri-dish in the dark. After one-week, pre-germinated rice seedlings were transplanted to soil and grown in a growth chamber with a day/night cycle of 12 h/12 h, 29 °C/26 °C with 350 μE m-2 s-1, and a relative humidity of 65%. Barley seeds (*Hordeum vulgare* L. ssp. vulgare, cultivar Commander) were directly sown onto soil and grown in a greenhouse at 21 °C. Arabidopsis thaliana (ecotype Columbia-0) seeds were surfaced-sterilized and stratified for 48 h at 4 °C. Plants were grown on soil in a growth chamber with a day/night cycle of 16 h/8 h at 22 °C (day)/19 °C (night) and 120 μE m-2 s-1.

Plants were sourced as follows: 1) *Arabidopsis thaliana* (Col-0) was obtained from NASC (NASC ID: N1092) and grown continuously in the Laboratory of Prof James Whelan. It was verified by Prof James Whelan from his seed stock registrar and confirmed by visual examination of plants that are grown for seed stocks. 2) *Oryza sativa* (Nipponbare) was obtained from Prof Huixia Shou, College of Life Science, Zhejiang University, from the Genetic Stocks Oryza (GSOR) stock centre (GSOR ID: 301164) and cultured for seed production annually and verified by Prof James Whelan. 3) *Hordeum vulgare* L (Commander) - Grain was obtained and verified by A/Prof Matthew Tucker after cultivation at the Waite Campus, University of Adelaide. Commander was bred at the University of Adelaide and is available from SeedNet (https://associatedgrain.com.au/wp-content/uploads/2014/11/Commander-Barley_Factsheet_2014.pdf). Commander is a malt-type barley that is well suited to all growing regions of Australia.

### Stress treatments, tissue collection, RNA isolation and qRT-PCR

Two-week-old Arabidopsis (growth stage: 1.06; Boyes et al., 2001), rice and barley (both growth stage 13; Zadok et al., 1974) seedlings were sprayed with 2 mM salicylic acid (SA), 1 mM methyl-viologen [54], 10 mM 3-Amino-1,2,4-triazole (3-AT), 100 μM abscisic acid (ABA) or 50 μM antimycin A with 0.01% Tween20 as a wetting agent until liquid dripped off the leaves. Spraying was repeated after 30 min. Mock control plants were treated in the same way with water and 0.01% Tween20. Leaf samples of rice and barley (1 cm middle segment of the top-most leaf) as well as whole rosette tissue for Arabidopsis were harvested and shock-frozen in liquid nitrogen for total RNA extraction. For UV treatment all plants were exposed to UVC irradiation (dominant wavelength 254) for 15 min in a sterilising hood with samples placed 10 cm from light source at RT using an OSRAM HNS 30 W G13 light source. The sample collection following all treatments was performed at the earliest time points for each treatment where the gene expression responses for the marker genes in Table S1 were observed by qRT-PCR or previously shown to be significant in rice or Arabidopsis, while for barley, the times tested were 3 h, 6 h and 12 h for the respective genes (Table S1). Those timepoints were 3 h after the initial spraying for all treatments in all species and treatments, except for AA and ABA in barley which was after 6 h. Treatment of rice seedlings with 50 μM AA was performed with 2 cm leaf segments floating in 10 mM potassium phosphate buffer (pH 6.8) with 0.01% Tween20 for 3 h. Mock treatment was conducted accordingly with water and 0.01% Tween20. Leaf samples were harvested and shock-frozen in liquid nitrogen for RNA-extraction. For total RNA isolation the tissue of 3–4 individual plants was pooled for each of the three biological replicates.

Total RNA was isolated using the SpectrumTM Plant Total RNA kit (Sigma-Aldrich) according to the manufacturer’s protocol. On-Column DNase I (Sigma-Aldrich) digestion was performed with the total RNA prior to elution. The quantity and quality of RNA was analysed using a SPECTROstar® (BMG LABTECH, Freiburg, Germany) spectrophotometer and agarose gel electrophoresis. For qRT-PCR analysis 1 μg of total RNA was reverse transcribed using the cDNA Synthesis Kit (Bio-Rad) according to the manufacturer’s instructions, further details in Supplemental materials.

### RNA-seq

RNA-seq libraries were prepared using the TruSeq Stranded mRNA Library Prep Kit according to manufacturer’s instructions (Illumina) and sequenced on a HiSeq1500 system (Illumina) as 60 bp reads with an average quality score (Q30) above 95% and on average 18 million reads per sample. Quality control was performed using the FastQC-software (https://www.bioinformatics.babraham.ac.uk/projects/fastqc/). Transcript abundances as transcripts per million (TPM) and estimated counts were quantified on a gene level by pseudo-aligning reads against a k-mer index build from the representative transcript models (TAIR10-Arabidopsis, IRGSP-1.0-rice, IBSCv2-barley) using the kallisto program with 100 bootstraps [140]. Only genes with at least 5 counts in a quarter of all samples per genotype were included in the further analysis. The program sleuth with a Wald test was used to test for differential gene expression [141]. Genes were called as differentially expressed with a false discovery rate FDR < 0.05 and a log2 (fold change) =/> 1.

### Bioinformatic analysis

Orthologues and corresponding orthogroups across Arabidopsis, rice and barley were inferred via OrthoFinder v. 2.3.3 [56] with default parameters and MMseqs2 [142] for sequence similarity searches. Protein sequences were retrieved from EnsemblPlants v44 (https://plants.ensembl.org/index.html) for barley (IBSCv2), TAIR (TAIR10) for Arabidopsis and IRGSP-1.0 for rice.

Hierarchical clustering and generation of heat maps was performed using the pheatmap R package [143].

Details of the TF enrichment analysis, Pageman analysis, GO-term analysis and motif analysis are in Supplemental methods.

## Supplementary Information

**Additional file 1: Supplemental Fig. 1a.** Functional categories of up-regulated genes in response to stress across species. **Supplemental Fig. 1b.** Functional categories of down-regulated genes in response to stress across species. **Supplemental Fig. 2.** Differentially expressed genes within orthogroups from Arabidopsis, rice and barley. **Supplemental Fig. 3.** Heatmap of MDS candidate genes in Arabidopsis. **Supplemental Fig. 4.** Expression of genes encoding transcription factors in Arabidopsis, rice and barley in response to different stress treatments. **Supplemental Fig. 5.** Enrichment of transcription factor families in response to stress across species. **Supplemental Fig. 6.** Transcription factor families that were enriched in oppositely responsive gene-sets (up/down-regulated).

**Additional file 2: Supplemental Table 1.** Gene expression of marker genes to validate stress treatments. **Supplemental Table 2.** Log2 FC of all differentially expressed genes in response to different stress treatments. **Supplemental Table 3.** Functional categories of differentially expressed genes in all species. **Supplemental Table 4**. Orthogroup inference in Arabidopsis, rice and barley and expression responses of DEGs within these. **Supplemental Table 5.** Conserved stress responses across species. **Supplemental Table 6.** Gene ontology (GO) analysis of differentially expressed Arabidopsis genes in response to stress. **Supplemental Table 7.** Gene expression pattern and the functional role of Arabidopsis genes. **Supplemental Table 8.** MDM enrichment analysis and identification of MDS candidate genes. **Supplemental Table 9.** Oppositely responsive genes in Arabidopsis, rice and barley following stress treatments. **Supplemental Table 10.** List of transcription factors in Arabidopsis, rice and barley. **Supplemental Table 11.** Enrichment analysis of all transcription factor families in response to stress in Arabidopsis, rice and barley. **Supplemental Table 12.** Oppositely responsive transcription factor families in different stress treatments. **Supplemental Table 13.** List of all qRT-PCR primer used in this study.

## Data Availability

All NGS data from this study has been submitted to GEO under the accession: Barley: https://dataview.ncbi.nlm.nih.gov/object/PRJNA655522?reviewer=8106fo6qrhll624dnds1plt36k Rice:https://dataview.ncbi.nlm.nih.gov/object/PRJNA655523?reviewer=eek2167n23ucnakjhn4gh9vj9o Arabidopsis: data is already public - PRJNA486068 [136].

## Abbreviations

3AT: 3-amino-1,2,4-triazole
AA: Antimycin A
ABA: Abscisic acid
AOX: Alternative oxidase
DEGs: Differentially expressed genes
ERF: Ethylene response factor
FDR: False discovery rate
GO: Gene ontology
HSF: Heat shock factor
JA: Jasmonic acid
NAC: NAM, ATAF, CUC transcription factor
NGS: Next-generation sequencing
MDM: Mitochondrial dysfunction motif
MDS: Mitochondrial dysfunction stimulon
MRR: Mitochondrial reterograde regulation
OG: Orthogroup
ROS: Reactive oxygen species
SA: Salicylic acid
TF: Transcription factor
MV: Methyl viologen
UV: Ultraviolet radiation
