# Supplementary material for: Cross-species transcriptomic analyses reveals common and opposite responses in Arabidopsis, rice and barley following oxidative stress and hormone treatment
Source: BMC Plant Biol. 2022 Feb 4;22:62. doi: 10.1186/s12870-021-03406-7 (PMC8815143; doi:10.1186/s12870-021-03406-7)
Supplement: Supplementary file 1 — Additional file 1: Supplemental Fig. 1a. Functional categories of up-regulated genes in response to stress across species. Supplemental Fig. 1b. Functional categories of down-regulated genes in response to stress across species. Supplemental Fig. 2. Differentially expressed genes within orthogroups from Arabidopsis, rice and barley. Supplemental Fig. 3. Heatmap of MDS candidate genes in Arabidopsis. Supplemental Fig. 4. Expression of genes encoding transcription factors in Arabidopsis, rice and barley in response to different stress treatments. Supplemental Fig. 5. Enrichment of transcription factor families in response to stress across species. Supplemental Fig. 6. Transcription factor families that were enriched in oppositely responsive gene-sets (up/down-regulated). [file 12870_2021_3406_MOESM1_ESM.pdf]

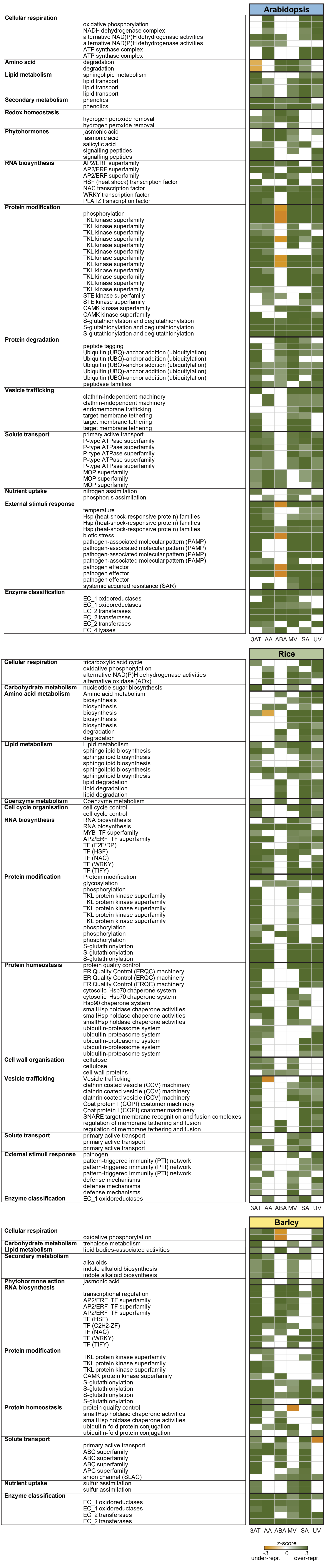

Supplemental Figure 1a. Functional categories of up-regulated genes in response to stress across species. Full PageMan (Usadel et al., 2006) output showing over- and under-represented (PageMan - Fisher's test) functional categories in all species for all differentially expressed genes that are upregulated. AA = antimycin A, ABA = abscisic acid, MV = methyl viologen, SA = salicylic acid, UV = ultraviolet radiation.

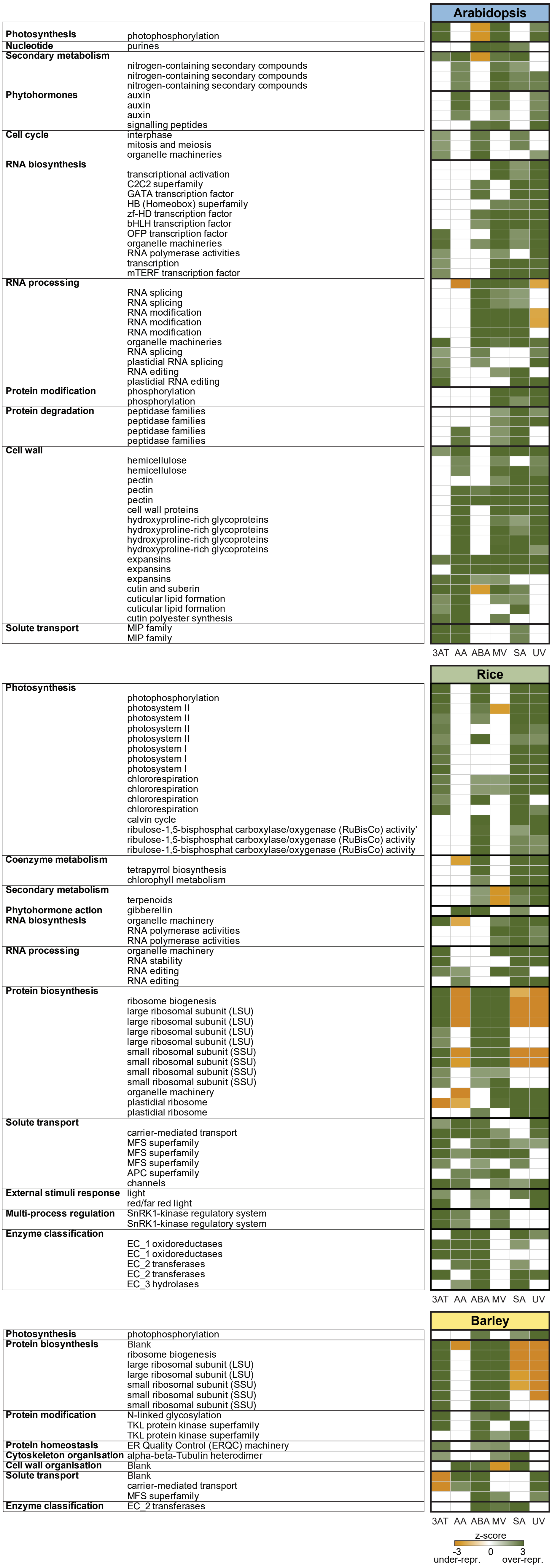

Supplemental Figure 1b. Functional categories of down-regulated genes in response to stress across species. Full PageMan (Usadel et al., 2006) output showing over- and under-represented (PageMan - Fisher's test) functional categories in all species for all differentially expressed genes that are upregulated. AA = antimycin A, ABA = abscisic acid, MV = methyl viologen, SA = salicylic acid, UV = ultraviolet radiation.

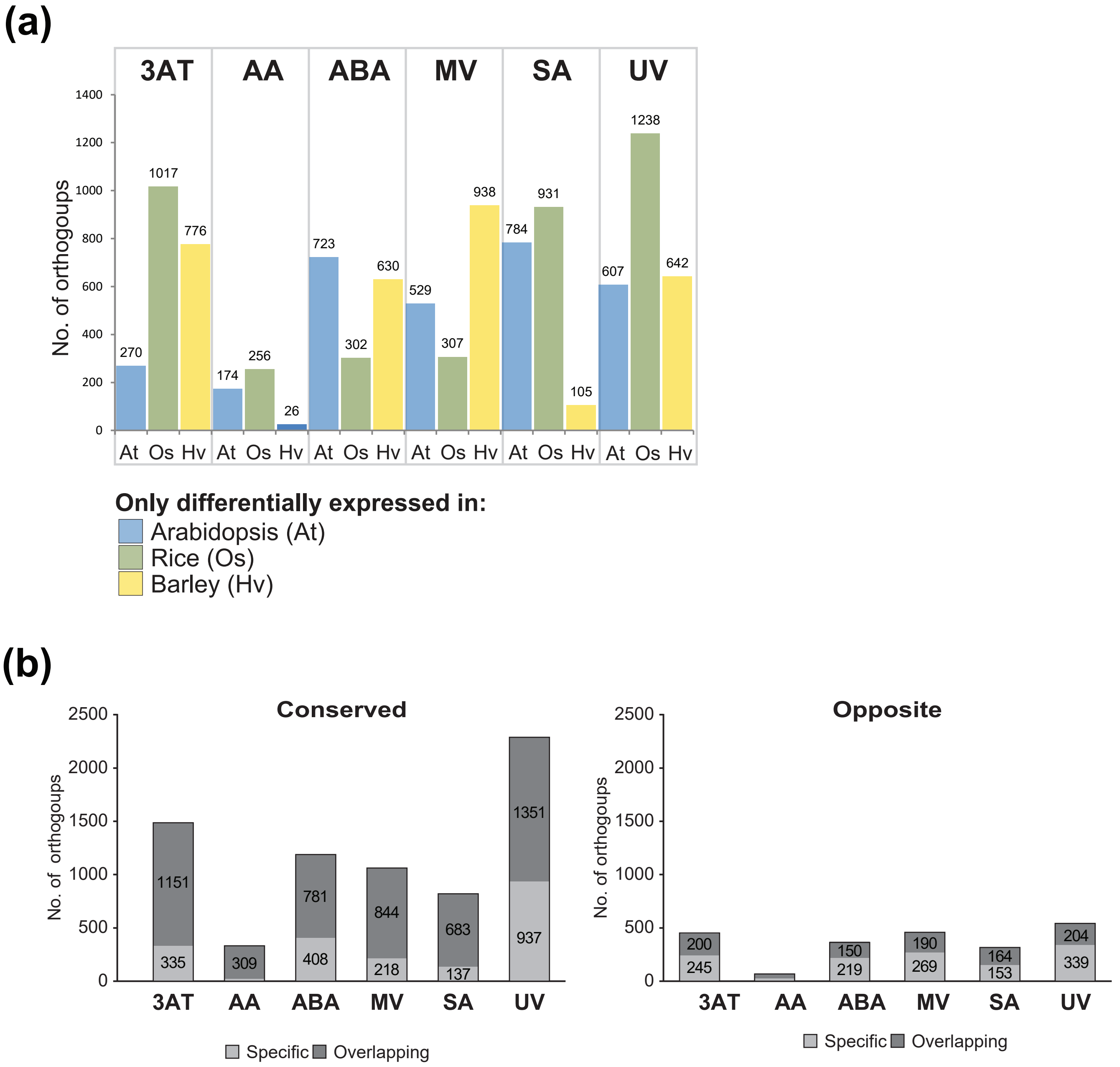

Supplemental Figure 2. Differentially expressed genes within orthogroups from Arabidopsis, rice and barley. a) The number of DEGs that were within orthogroups in which significant differntial expression was only observed in one species, while the ortholgous genes in the other species within that orthogroup remain unchanging in expression. b) Conserved and distinct stress responses in Arabidopsis, rice and barley. The total number of conserved and opposite OGs for each stress with the numbers that were treatment specific (light grey) and overlapping (dark grey) indicated. 3AT = 3-amino-1,2,4-triazole; AA = antimycin A; ABA = abscisic acid; MV = methyl viologen; SA = salicylic acid; UV = ultraviolet radiation; At = Arabidopsis; Os =Rice; Hv = Barley.

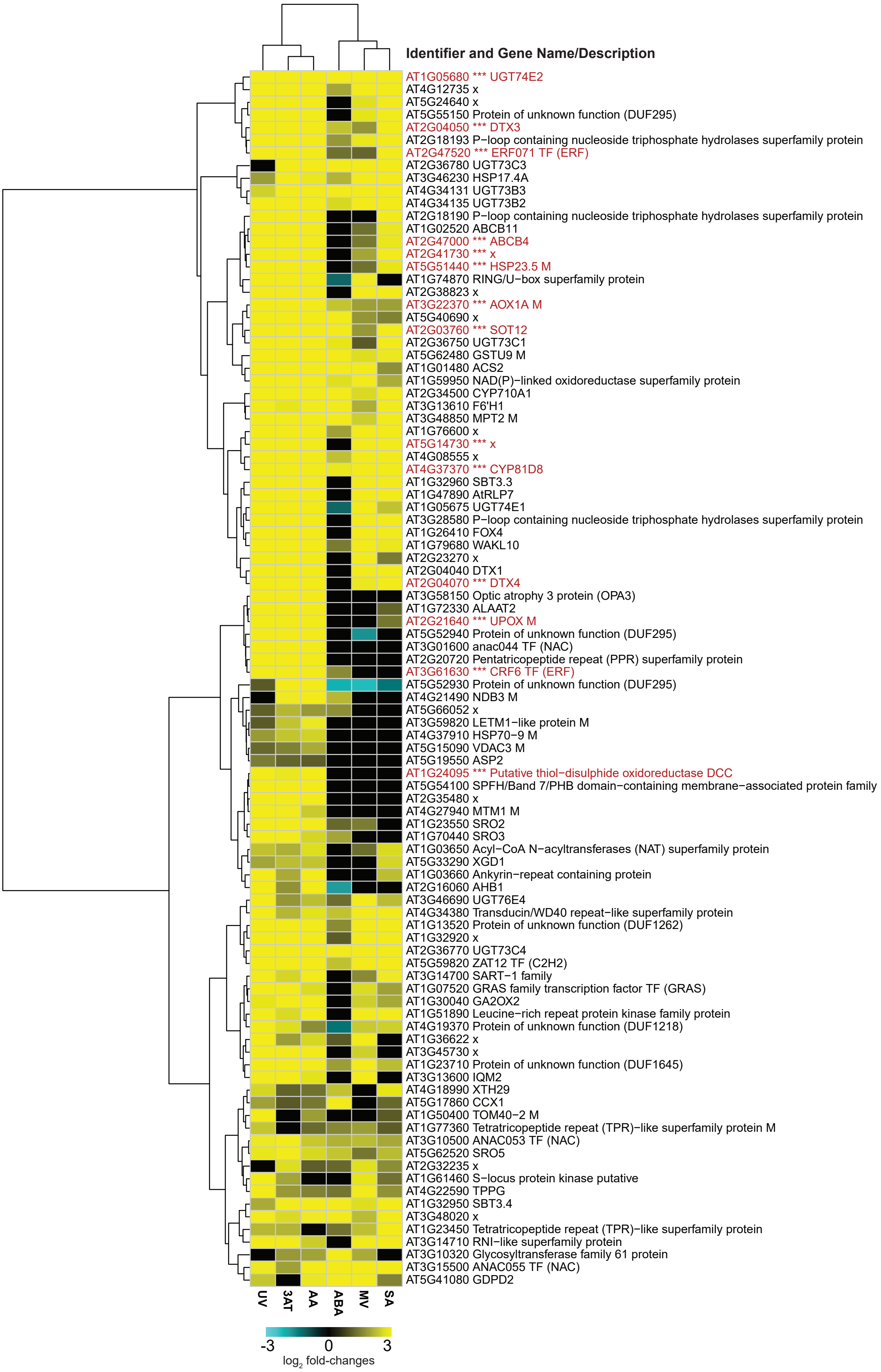

Supplemental Figure 3. Heatmap of MDS candidate genes in Arabidopsis. Hierarchically clustered heatmap of expression values from all ANAC017 gene regulatory network (GRN) genes (Meng et al., 2019) that containing the stringent MDM in their promotor region, are highly stress responsive to AA and or 3AT and differentially expressed in at least 3 out of 6 treatments. Previously characterised MDS genes (De Clercq et al., 2013) are highlighted in red. AA = antimycin A, ABA = abscisic acid, MV = methyl viologen, SA = salicylic acid, UV = ultraviolet radiation.

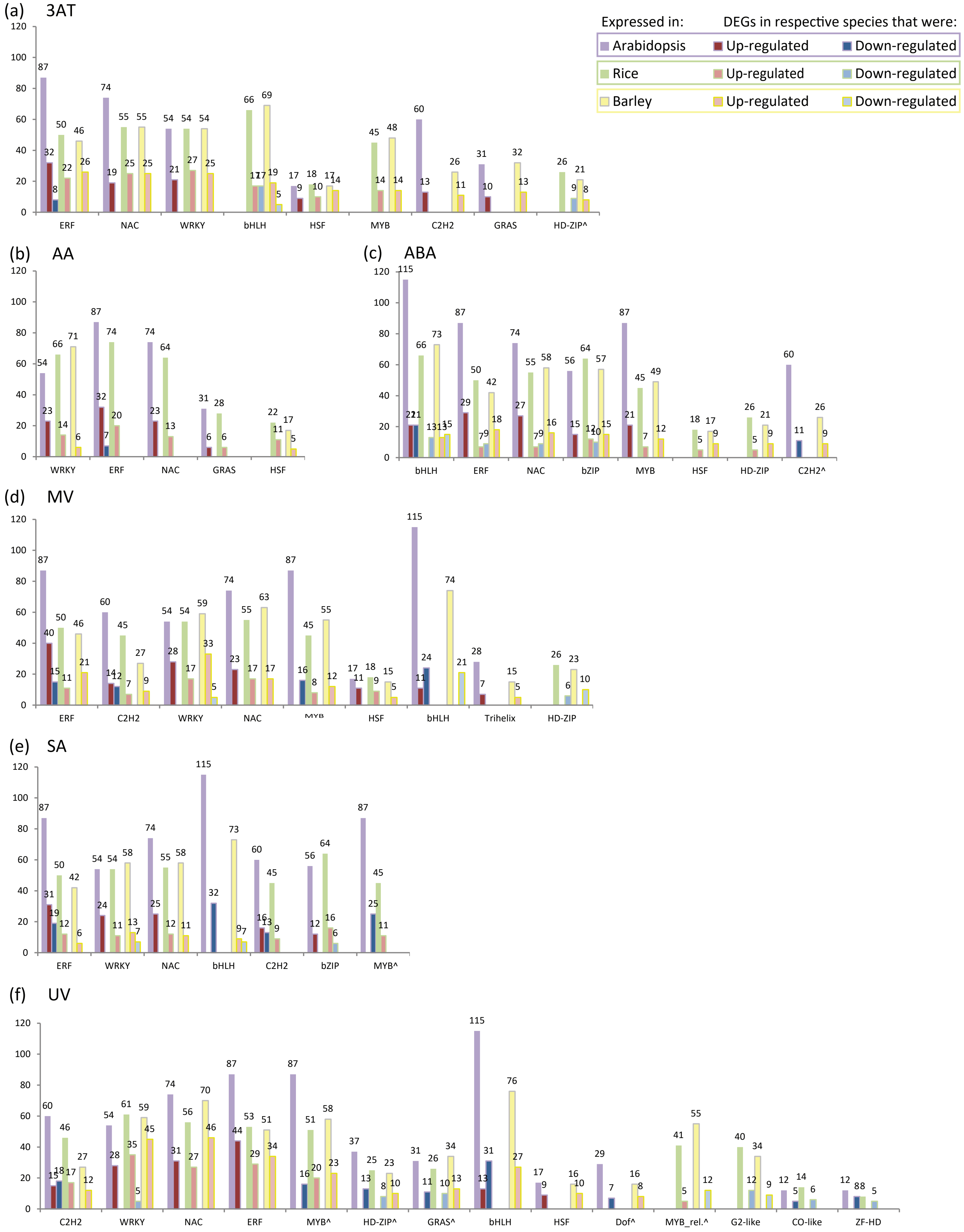

Supplemental Figure 4. Expression of genes encoding transcription factors in Arabidopsis, rice and barley in response to different stress treatments.
Numbers of transcription factors (TFs) expressed in the TF families in Arabidopsis (purple), Rice (green) and Barley (yellow) are shown, next to columns indicating the number that were up-regulated (red/pink) or down-regulated (blue/light blue) for that family. (a) 3AT = 3-amino-1,2,4-triazole, (b) AA = antimycin A, (c) ABA = abscisic acid, (d) MV = methyl viologen, (e) SA = salicylic acid, (f) UV = ultraviolet radiation. The number of TFs expressed in the families that were enriched in at least two species across at least two stresses. Note ^ indicates families that are over-represented in oppositely responsive sub-sets.

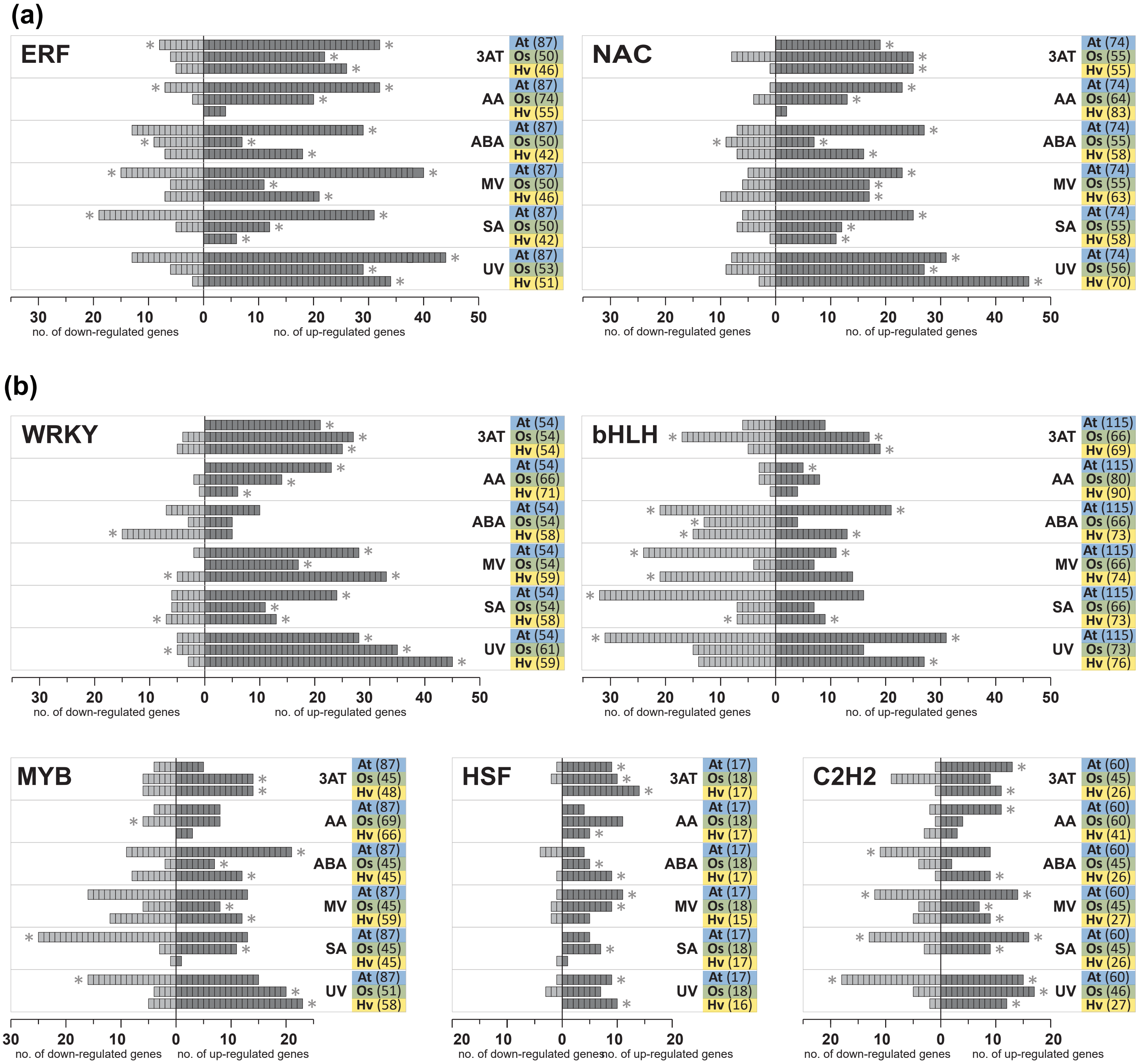

Supplemental Figure 5. Enrichment of transcription factor families in response to stress across species.
Expression of genes encoding transcription factors (TFs) in Arabidopsis (At), rice (Os) and barley (Hv) in response to different stress treatments. a) Number of differentially expressed (up-regulation = dark grey; down-regulation = light grey) TFs with families that were enriched (hypergeometric distribution; p-value< 0.05; Supplemental Table 11) in at least two species across all six stresses. ERF and NAC TF families are visualized. The total number of TFs identified for each family in each species (http://planttfdb.cbi.pku.edu.cn/) is shown in brackets b) Number of differentially expressed TFs with families that were enriched in at least two species across five stresses. WRKY, bHLH, MYB, HSF and C2H2 TF families are visualized. 3AT = 3-amino-1,2,4-triazole, AA = antimycin A, ABA = abscisic acid, MV = methyl viologen, SA = salicylic acid, UV = ultraviolet radiation.

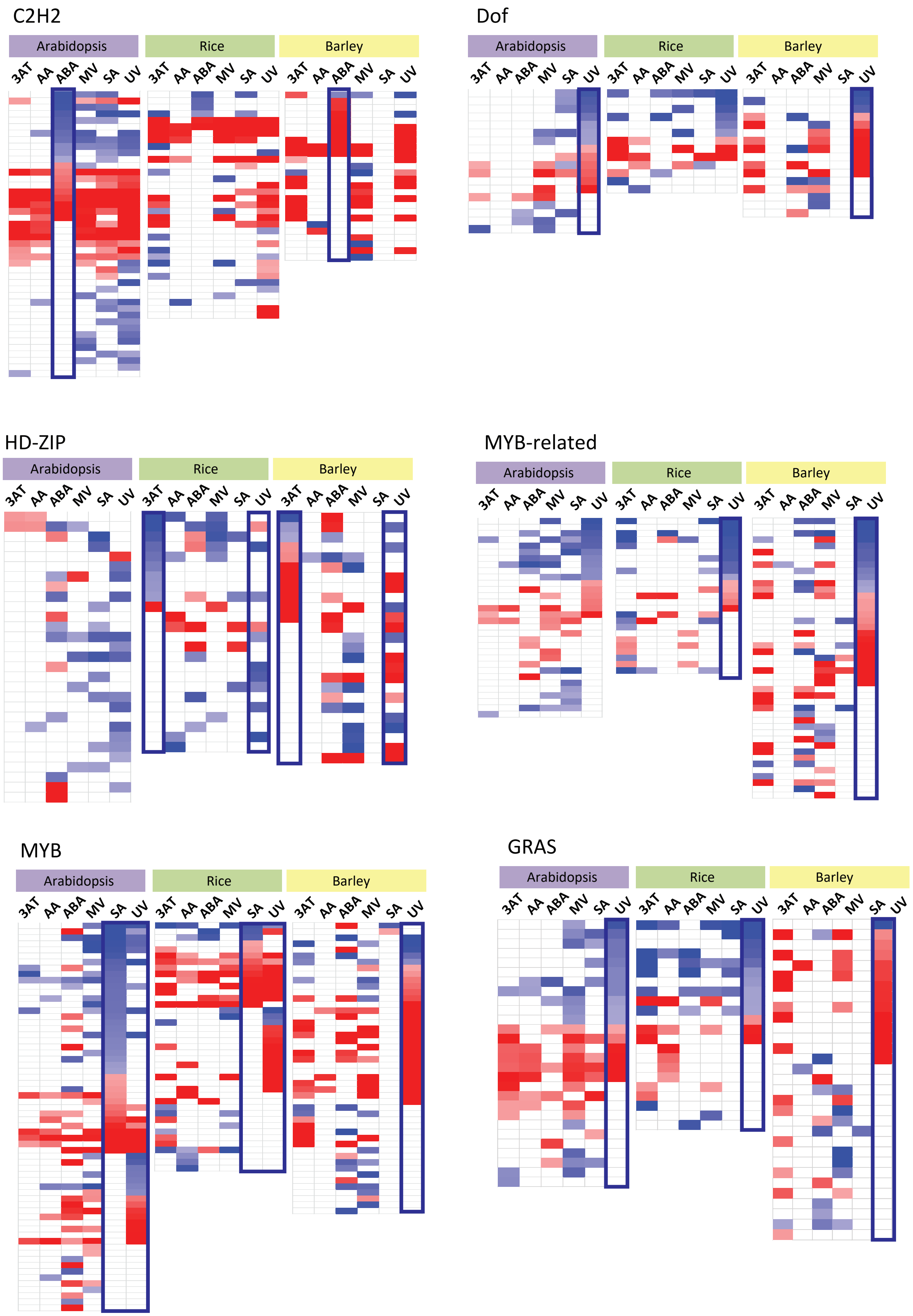

Supp Fig 6. Transcription factor families that were enriched in oppositely responsive gene-sets (up/down-regulated). Heatmaps showing gene expression of different transcription factor families that show opposite responses to some treatments in Arabidopsis, rice and barley. 3AT = 3-amino-1,2,4-triazole, AA = antimycin A, ABA = abscisic acid, MV = methyl viologen, SA = salicylic acid, UV = ultraviolet radiation.
